# A principal-stress rule for cell division in epithelia

**DOI:** 10.1038/s41467-026-77813-5

**Published:** 2026-09-23

**Authors:** Lucas Anger, Tianxiang Ma, Fanny Wodrascka, Andreas Schoenit, Kristian Thijssen, Satish Kailasam Mani, Marc-Antoine Fardin, Carine Rossé, René-Marc Mège, Amin Doostmohammadi, Benoit Ladoux

**Affiliations:** 1 Université Paris Cité, CNRS, Institut Jacques Monod, F-75013 Paris, France; 2 Niels Bohr Institute, University of Copenhagen, Copenhagen, Denmark; 3 Institut Curie, Paris Université Sciences et Lettres, Sorbonne Université, CNRS, UMR144, Paris, France; 4 Department of Physics, Friedrich-Alexander-Universität Erlangen-Nürnberg, Erlangen, Germany; 5 Max-Planck-Zentrum für Physik und Medizin and Max Planck Institute for the Science of Light, Erlangen, Germany

**Keywords:** Biophysics, Cell biology

## Abstract

Dense active materials must continuously relieve internal mechanical stress to remain structurally stable as they are driven far from equilibrium. In epithelial tissues, this relief occurs through cell division, where, according to Hertwig’s century-old rule, cells divide along their long axis. We show that as epithelia densify, this geometric rule is superseded by a mechanical one: cells divide along the principal axis of anisotropic stress, independently of the isotropic stress state. Using direct force measurements and stress inference, we demonstrate that stress orientation governs division across mechanically distinct systems, from fluid-like to jammed monolayers and structurally heterogeneous organoids, remaining predictive where cell shape fails. This stress-oriented remodeling is reciprocally coupled to the material’s mechanical state: anisotropic stress accelerates cell-cycle progression, while division locally dissipates it, closing a negative feedback loop. This “principal-stress rule” provides a general mechanical framework linking internal stress, structural remodeling and homeostasis in living materials.

## Abstract (web summary)

The search for general physical principles that govern biological organization has long motivated studies of tissue mechanics. In this work, the authors show that a simple mechanical rule based on principal stress predicts the orientation and rate of cell division across dense epithelial systems, revealing cell division as a key mechanism for stress regulation in living matter.

## Introduction

Cell division is a central process in development, homeostasis, and disease, and its regulation has long been studied from mechanical perspectives. Classic experiments dating back to the 19th century led to Hertwig’s rule, which states that cells orient their division along their long axis, reflecting the geometry of interphase shape^1,2^. This geometric principle has since been refined in diverse contexts, including epithelial layers where cell shape and mitotic spindle alignment appear coupled^3–6^.

Beyond geometry, accumulating evidence points to a central role for forces and stresses. Tension has been shown to accelerate proliferation^7–9^, compression can inhibit it^10–12^, and external forces can orient spindles independently of cell shape^13^. Within tissues, cells have also been observed to divide under compression or in mechanically ambiguous states, raising questions about whether shape or stress provides the dominant physical signal^14–20^. Despite extensive research, a unified framework reconciling geometry and stress-based control of cell division has remained elusive.

Dense epithelial tissues provide a particularly stringent test. As cells proliferate and crowd, they round up and adopt isotropic shapes^21–23^. In this regime, orientational order defined by cell shape or actin organization collapses, leaving Hertwig’s geometric rule no longer a reliable predictor. Yet tissue-level regulation of divisions must continue^24,25^, suggesting that a more general principle must govern their orientation.

Here we show that in dense epithelia, division is governed not by geometry but by mechanics. Cells align their division axis with the direction of maximal anisotropic stress, a “principal-stress rule” that generalizes Hertwig’s principle to tissues where shape cues fail, while remaining equally predictive when such cues are present. Notably, this principle is independent of the isotropic stress state, as mechanical anisotropy can exist even in the absence of a net isotropic stress. We find across different cell types and varying cell densities that stress orientation robustly governs division orientation, even as tissues jam and cell elongation disappears. Moreover, anisotropic stress not only predicts division orientation but also accelerates the cell cycle, while cytokinesis in turn dissipates anisotropy. This establishes a feedback loop that links stress, division, and homeostasis within a unified mechanical framework.

## Results

### A principal-stress rule extends Hertwig’s long-axis rule

**Fig. 1:**
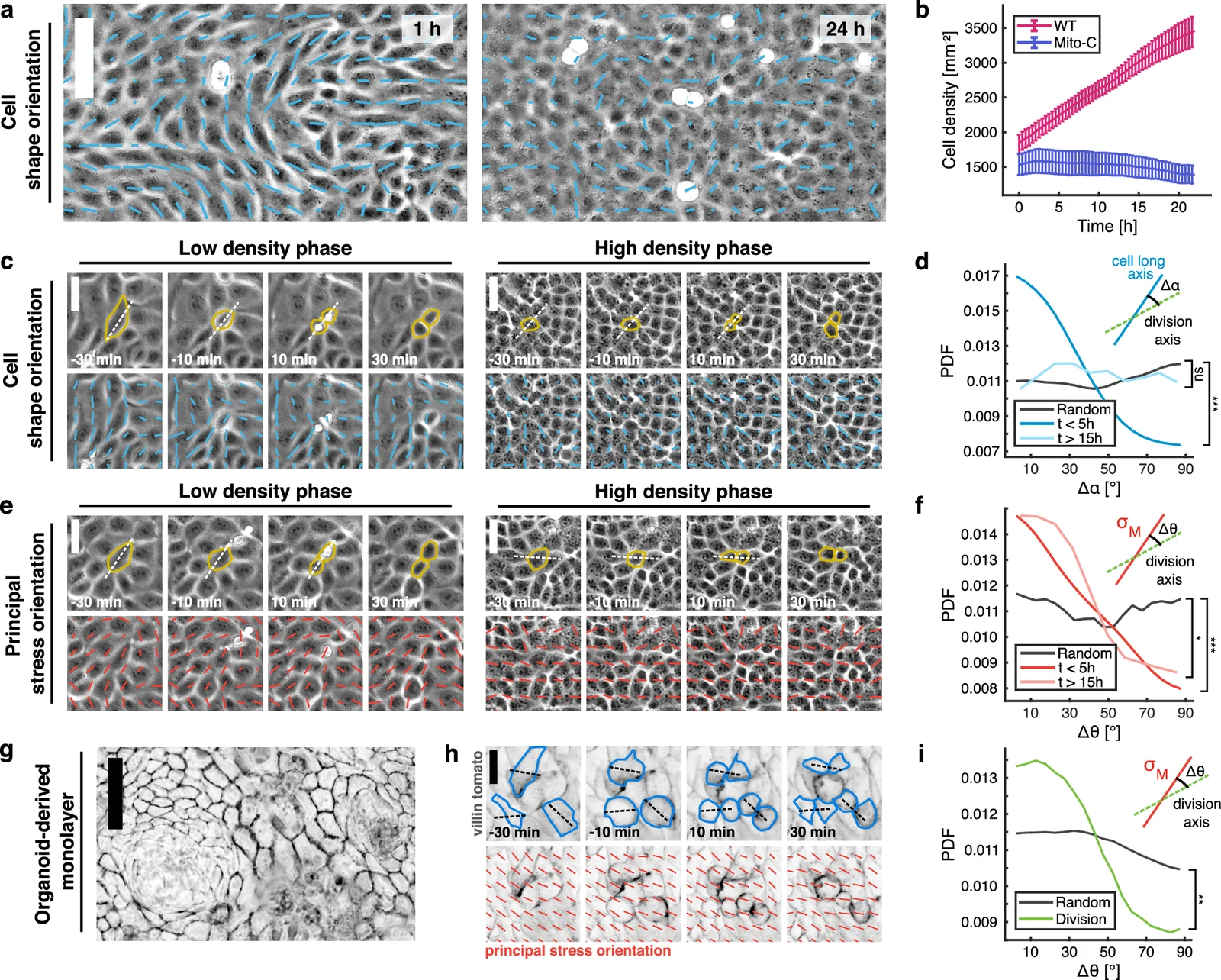
Principal stress orientation predicts cell division orientation throughout tissue densification. **a** Left, phase contrast image of confluent MDCK monolayer in low density state, right, in high density state. Nematic director in blue. **b** Mean evolution in time of the cell density, for MDCK WT and for mitomycin-C treated MDCK cells. *n* = 15 movies from *N* = 3 independent experiments (WT) ; *n* = 15 movies from *N* = 3 independent experiments (Mito-C). **c** Phase contrast image of cell division process, showing in white line cell division orientation, before and after cytokinesis for (left) a low density MDCK monolayer and for (right), high density. Nematic director in blue. **d** PDF of the angle between MDCK cell division orientation and nematic director orientation for different cell density. 681 cell divisions (t  < 5h), 234 cell divisions (t  > 15h), 1690 random orientations at random positions. *n* = 13 movies from *N* = 3 independent experiments. *p*  = 2.107e-16 (t  < 5h vs random) and *p*  = 0.197 (t  > 15h vs random). **e** Phase contrast image of cell division process, showing in white line cell division orientation, before and after cytokinesis for (left) a low density MDCK monolayer and for (right), high density. Stress orientation in red. **f** PDF of the angle between MDCK cell division orientation and stress orientation for different cell density. 681 cells divisions (t  < 5h), 234 cells divisions (t  > 15h), 1690 random orientation at random position. *n* = 13 movies from *N* = 3 independent experiments. *p*  = 1.154e-6 (t  < 5h vs random) and *p*  = 0.0299 (t  > 15h vs random). **g** Phase contrast image of intestinal organoid-derived monolayer after being cultivated for three days. **h** Confocal images of cell division process, showing in black line cell divisions orientations, before and after cytokinesis for villin-tomato intestinal organoid monolayer. Stress orientation in red. **i** PDF of the angle between organoid cell division orientation and stress orientation. 620 cell divisions and 1950 random orientation at random position. *n* = 13 movies from *N* = 3 independent experiments. *p*  = 0.0022 (stress vs random). [Scale bar 80 μm (**a**, **g**), 20 μm (**c**, **e**, **h**). *P*-value from two-sided Mann-Whitney U-test (**d**, **f**, **i**). All error bars show the confidence interval at 95 %.].

For over a century, Hertwig’s long-axis rule has been the dominant paradigm for understanding division orientation^1,2^. We first revisited this rule in various types of epithelial monolayers including cell lines and intestinal organoids. Starting with Madin-Darby Canine Kidney (MDCK) cells, we observed that at low density cells were elongated (Fig. 1a, b) and divisions aligned with the long axis, consistent with both the classical rule and modern refinements linking spindle positioning to geometry^3,4,6^ (Fig. 1c, d). This served as an internal validation that our system recapitulates established principles.

As confluent epithelial tissues densified, however, cell geometry changed dramatically: elongated shapes gave way to isotropic, hexagonally packed cells (Fig. S1a–d), consistent with previous studies^26,27^. In this regime, the long axis is weakly defined (Fig. S1e-f, Movie S1), and divisions are uncoupled from cell shape, indicating that a cue other than geometry must dominate (Fig. 1c, d). The breakdown of Hertwig’s rule in this regime highlights a conceptual gap: while geometric alignment explains orientation in sparse epithelia, it fails in precisely those crowded contexts where control of division orientation is most consequential for tissue architecture^19,24,28^.

If geometry cannot explain division orientation in dense tissues, what alternative cue does? A natural candidate for such a cue is mechanics. Cells continuously generate, transmit, and respond to forces^29,30^ that can, in principle, provide directional information. We therefore asked whether local stress fields, rather than geometry, might provide the directional information that persists when shapes become isotropic. To test whether division orientation reflects stress rather than shape, we first measured the traction forces exerted by cells on their substrates (Fig. S2a, b) and subsequently inferred the stresses using Bayesian inference stress microscopy^31,32^ (Fig. S2c–e, Movie S2). At any given point of space within the epithelial sheet, the local in-plane coordinate system can be rotated to identify the specific directions along which the normal stress reaches its maximum and minimum values^33^ (Fig. S2f). These directions define the maximal principal stress, *σ*_*M*_, the minimal principal stress, *σ*_*m*_ and the principal stress orientation *θ* (see Supporting Information). Physically, this manipulation corresponds to separating the stress tensor into isotropic and deviatoric components, whose invariants define the isotropic stress $\frac{{\sigma }_{M}+{\sigma }_{m}}{2}$ (Fig. S2c-d) and the anisotropic stress $\frac{{\sigma }_{M}-{\sigma }_{m}}{2}$, the latter being applied along the principal stress direction (Fig. S2e–g). Interestingly, temporal autocorrelation analysis indicated that principal stress orientation was slightly more temporally stable than shape anisotropy (Fig. S2h).

Across densities, division orientation was consistently aligned with the principal stress axis (Fig. 1e, f, Fig. S3a). Importantly, this “principal-stress rule” also held in intestinal epithelial organoids^34,35^, despite their heterogeneous distributions of cell types, shapes, and sizes (Fig. 1g–i, Fig. S3b, Movie S3). Finally, to test the generality of our findings, we examined epithelial cell lines covering a range of mechanical behaviors, from fluid-like mammary gland MCF10A cells (Fig. S3c–e), in which elongation persists over long times^36^, to solid-like N/TERT1 keratinocytes (Fig. S3f–h)^37^, which jam early and do not exhibit elongated shapes. Across these diverse mechanical contexts, including tension, compression, fluid-like and jammed regimes, and the mixed cell-type monolayers of intestinal organoids, stress orientation consistently provided a robust predictor of the division axis.

**Fig. 2:**
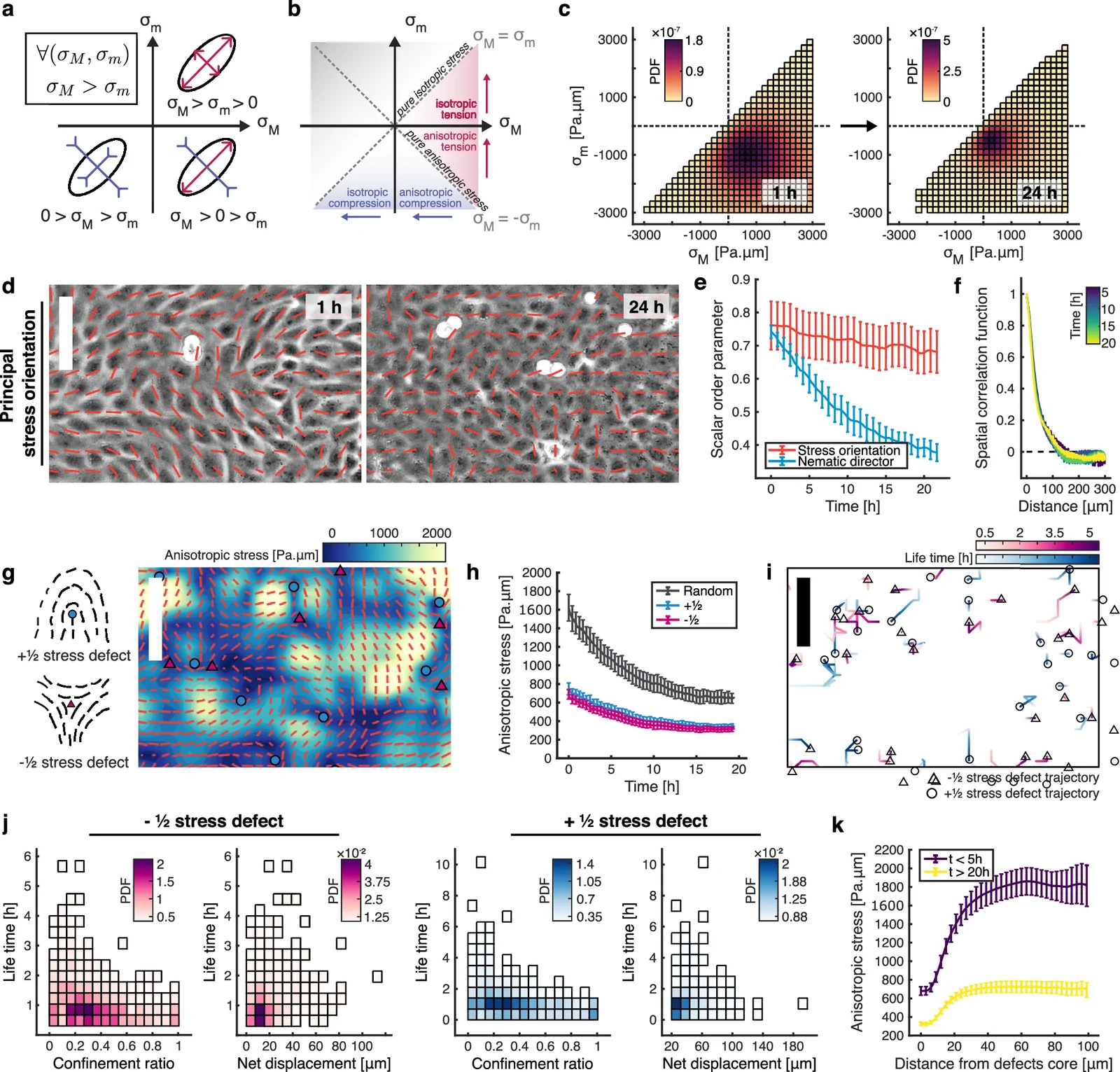
Anisotropic stress defines tissue mechanical landscape in space and in time. **a** Mechanical state diagram illustrating all possible coupling between maximum and minimum principal stress. **b** Detailed diagram corresponding to **a**. Red is tension, blue is compression, gray area is prevented by construction. **c** 2D histogram of the maximum and minimum principal stress coupling within MDCK monolayer, left, at low density, and right, at high density. Color code is the PDF. *n* = 24 movies from *N* = 5 independent experiments. **d** Phase contrast image of MDCK monolayer at low density (left), and at high density (right). Stress orientation field in red. **e** Evolution of the apparent scalar order parameter for the nematic director (blue) and for the stress orientation (red) in time. *n* = 15 movies from *N* = 3 independent experiments. **f** Decay of the spatial correlation function of the stress orientation for various moments in time. Color code is the time. *n* = 15 movies from *N* = 3 independent experiments. **g** Left, cartoon of various topological stress defects charge possible, right, anisotropic stress field snapshot within the monolayer (t = 10h). Stress orientation in red. **h** Anisotropic stress evolution during densification around stress defects and random positions. *n* = 24 movies from *N* = 5 independent experiments. **i** Representative stress defects trajectory within a 5h time window. **j** 2D histogram of stress defects lifetime regarding, left, confinement ratio and right, net displacement. $+\frac{1}{2}$ stress defects in blue (*n* = 2358), $-\frac{1}{2}$ stress defects in pink (*n* = 1897). *n* = 5 movies from *N* = 5 independent experiments. Color code is the PDF. **k**) Average anisotropic stress evolution regarding distance from defects core, regardless of the defect charge. *n* = 24 movies from *N* = 5 independent experiments. [Scale bar 80 μm (**d**, **g**, **i**). All error bars show the confidence interval at 95 %. $+\frac{1}{2}$ defects in blue circle, $-\frac{1}{2}$ defects in pink triangle.].

This generality is key. Prior reports noted cases where cells divided orthogonally to their shape, along an anisotropic tension or perpendicular to mechanical compression^3,14–17,19,28,38^, but lacked a unifying principle. In all experimental conditions analyzed in this study, divisions are aligned with the stress axis. This principal stress orientation can emerge under compressive, tensile, or even zero isotropic stress conditions (Fig. 2a, b). In our data, tissue mechanical anisotropy is created under both compression or tension, and remained systematically non-zero during monolayer densification, thereby defining a stress orientation (Fig. 2c and Fig. S3a-b, e, h). Thus, the principal-stress rule is a general framework: even if geometric cues vanish, stress anisotropy continues to orient divisions and extends Hertwig’s rule regardless of the isotropic stress state.

### Stress orientation persists when orientational order collapses

Building on the stress metrics defined above, we next investigated how stress orientations evolve over time. Indeed, prior studies have revealed that cellular monolayers could display orientational order, such as nematic domains arising from cell shape or actin alignment^26,39,40^, which by proxy would be expected to influence division orientation through Hertwig’s rule. We therefore compared how shape-based versus stress-based order evolve as tissues densify. At low density, MDCK monolayers exhibited nematic domains spanning hundreds of microns, with actin fibers aligned across cells (Fig. S4a). These orientational cues coincided with division alignment, consistent with the idea that collective order provides the directional bias. With increasing cell density, however, nematic order collapsed and cells adopted isotropic packing (Fig. S4a-d). This loss occurred across scales, single-cell actin ordering also disappeared at high density, confirming that orientational cues derived from geometry or cytoskeletal alignment vanish in crowded epithelia (Fig. S4d, e), without being replaced by an apico-basal anisotropy arising from mismatches between the apical and basal cell surfaces (Fig. S4f).

In contrast, stress orientation remained highly ordered. Even in jammed tissues^41^, where motion slowed and shape order was lost (Fig. S5a-d), stress domains persisted with strong alignment (Fig. 2d, e). Importantly, the scale of stress domains did not shrink with density; rather, anisotropy extended over 5–6 cell diameters (Fig. 2f). This persistence explains why division orientation remains coherent even as local geometry becomes isotropic (Movie S4). To further probe the limits of stress orientation in our system and test its quantification by BISM, we imposed specific stress patterns using controlled boundary conditions via micropatterning^42^. Confining cells to elliptical patterns generated a principal stress orientation along the major axis, along which cells preferentially divided (Fig. S6a, b). Consistently, laser ablation induced recoil predominantly along the major axis (Fig. S6c–e), independently validating both the ability of epithelia to adopt a collective anisotropic stress organization and the ability of BISM to recover the orientation of the deviatoric stress tensor^32^.

These results highlight an essential distinction: geometric order is unstable, collapsing under crowding, whereas stress order is robust, integrating forces across many cells. Stress anisotropy therefore provides a global orientational field that is insulated from local packing fluctuations. In dense tissues, where reliable alignment is needed to prevent uncontrolled anisotropy^43^, stress-based order emerges as the dominant organizing principle.

Beyond persistent order, stress fields also exhibited distinct topological defects. Unlike shape- or actin-based nematic defects, which disappeared as density increased, defects in the stress field persisted even in jammed monolayers (Fig. 2g and Fig. S7a-b) and remained spatiotemporally stable (Fig. 2h-j and Fig. S7c-f). To test whether these structures influence proliferation, we measured the spatial distribution between defects and division events. Divisions occurred at an average distance of  ~ 35μm from the nearest defect, nearly half the mean spacing between defects of the same type ( ~ 65μm, Fig. S8a-c). This indicates that divisions do not occur at defect cores, but preferentially at intermediate distances where stress anisotropy is elevated (Fig. 2k and Fig. S8d-e) and stress orientation is well defined. In this sense, defects act as organizing centers that spatially repel divisions while promoting them in surrounding high-anisotropy regions.

### Anisotropic stress regulates cell cycle progression

**Fig. 3:**
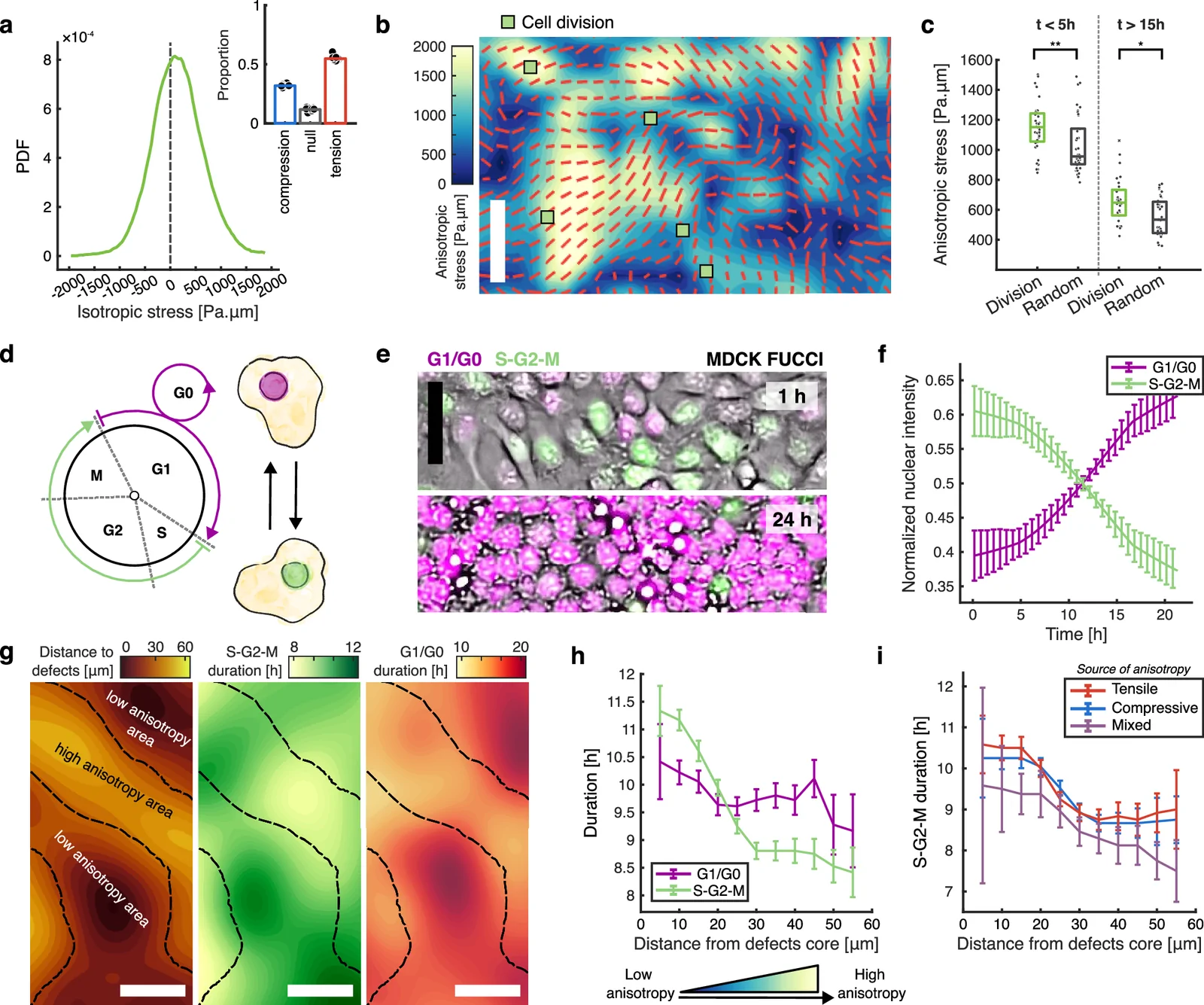
High anisotropic stress level modulates cell cycle completion. **a** PDF of the isotropic stress around cell division right before cytokinesis. 1672 cell divisions. Corresponding discrete barplot of the proportion of each mechanical state (compression, *σ*_*iso*_ < − 100, tension, *σ*_*iso*_ > 100, and null,  − 100 < *σ*_*iso*_ < 100) is also shown. *n* = 13 movies from *N* = 3 independent experiments. **b** Anisotropic stress field snapshot within the monolayer (t = 5h). Stress orientation in red. Cell divisions happening within a 1h window are marked in green square. **c** Average anisotropic stress around cell division localization at the moment of cytokinesis and random position during densification. 583 divisions (t  < 5h), 142 divisions (t  > 15h), 266 random positions (t  < 5h) and 387 random positions (t  > 15h). 39 points, *n* = 13 movies from *N* = 3 independent experiments. p = 0.0039 (t  < 5h) and *p* = 0.0049 (t  > 15h). **d** Simplified sketch of the FUCCI MDCK cell line principle. **e** Top, phase contrast image of MDCK FUCCI monolayer at low density, bottom, at high density. **f** Evolution in time of the S-G2-M cells to total cell ratio and G1/G0 cells to total cell ratio during densification. *N* = 10 movies from *n* = 2 independent experiments. **g** Left to right in order, temporal average of the distance from defects core map, corresponding map of the S-G2-M duration, projected in time, and corresponding map of the G1/G0 duration, projected in time. The black line corresponds to the isoline of a distance to defects of 31 μm. **h** Cell cycle phase duration regarding distance from stress defects core. *N* = 14 movies from *n* = 3 independent experiments. **i** Cell cycle S-G2-M phase duration depending on the local anisotropic stress generation (blue, compressive anisotropy, red tensile anisotropy, mix of tensile and compressive anisotropy) regarding distance from stress defects core. *N* = 14 movies from *n* = 3 independent experiments. [Scale bar 50 μm (**b**, **e**, **g**). *P*-value from two-sided Mann-Whitney U-test (**c**). All error bars show the confidence interval at 95 %.].

While orientation determines where cells divide, proliferation also depends on when. Mechanical signals have long been implicated in proliferation^44^, with tension reported to promote cell cycle entry^7,8^ and compression to inhibit it^10–12^. However, these models focus on isotropic stress, leaving open whether anisotropy itself plays a role. In our experiments, nearly 40% of divisions occurred in compressed or isotropically neutral regions (Fig. 3a), inconsistent with a purely tension-based explanation. Instead, it was high anisotropy, the directional imbalance of stress, that correlated with more cytokinesis events (Fig. 3b, c).

Using MDCK FUCCI cells, we tracked cell cycle phases during tissue densification. As expected, increasing density correlated with more cells in G1/G0 phases, consistent with contact inhibition of proliferation (Fig. 3d–f, Movie S5). Yet local stress measurements revealed a striking pattern: cells in regions of high anisotropy exhibited shortened S-G2-M phases, resulting in faster cell cycle progression (Fig. 3g, h). This effect was observed regardless of the nature of the stress anisotropy, whether tensile, compressive, or a mixture of both. (Fig. 3i and Fig. 2a, b).

These results revise our understanding of mechanical regulation of proliferation. Rather than acting as a simple on-off switch based on scalar pressure, cells appear to respond to the degree of directional imbalance. Moreover, the results show that anisotropic stress can regulate the cell cycle regardless of the state of its isotropic counterpart. Importantly, these results do not contradict previous work, since isotropic stress can be associated with an anisotropic stress organization^8,33^. This reframing resolves contradictions in the literature and positions anisotropy as a primary mechanical regulator of cell division.

### Cell division releases stress anisotropy through a feedback loop

If anisotropic stress promotes proliferation, how does the tissue avoid never-ending growth? Cell contact inhibition of proliferation is a well established mechanism in epithelia^11,45^, and increased cell density is often associated with elevated compressive stress^10,12,44^. In our data however, we did not observe an increase of isotropic compressive stress with tissue densification (Fig. S9a–c). Thus, we hypothesized that divisions themselves act back on the stress anisotropy, closing a feedback loop^15,46^.

**Fig. 4:**
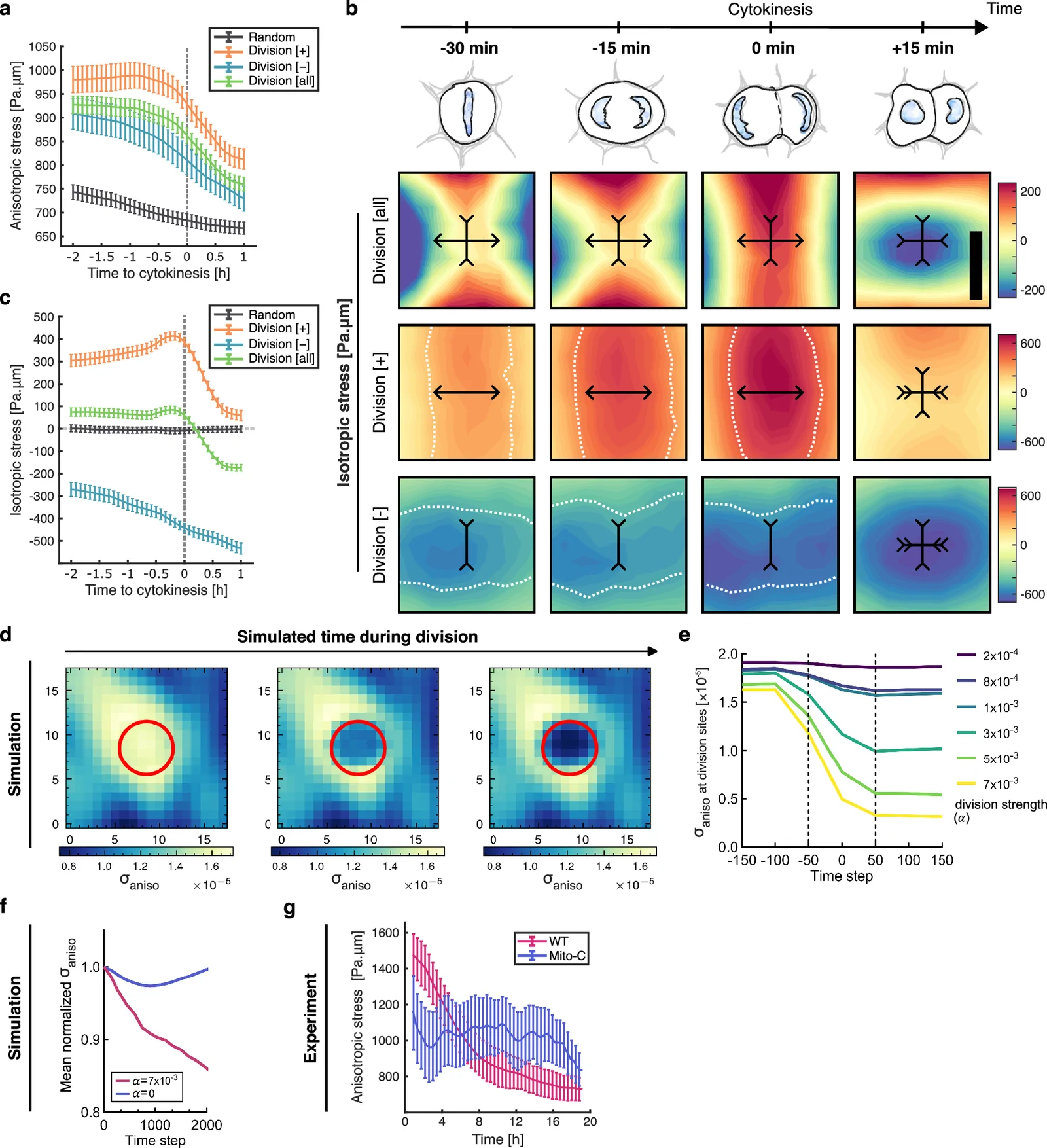
Cell division events release stress anisotropy during densification. **a** Average anisotropic stress around cell undergoing mitosis. Cytokinesis happens at t = 0h. The legend show the average for random position, for division events undistinguished ([all]) and for sub-distribution of cells dividing under tension ([+]) and cell dividing under compression ([-]). 1672 cell divisions, 498 cell divisions [-], 826 cell divisions [+] and 2219 random positions. *n* = 13 movies from *N* = 3 independent experiments. **b** Isotropic stress field around cell division before and after cytokinesis, oriented along the cell division axis. The black double arrow show the orientation of the maximum and minimum stress, with their sign. top, 1478 cell divisions [all], middle 690 cell divisions [+], and bottom 457 cell divisions [-]. *n* = 13 movies from *N* = 3 independent experiments. **c** Average isotropic stress around cell undergoing mitosis. Cytokinesis happens at t = 0h. The legend show the average for random position, for division events undistinguished ([all]) and for sub-distribution of cells dividing under tension ([+]) and cell dividing under compression ([-]). 1672 cell divisions [all], 498 cell divisions [-], 826 cell divisions [+] and 2219 random positions. *n* = 13 movies from *N* = 3 independent experiments. **d** Simulated spatial maps of anisotropic stress before, during, and after a localized division event. The red circle indicates the site of division. **e** Temporal evolution of anisotropic stress at the division site in simulations with varying division strengths (*α*). Black dashed lines denote the division period (Δ*t* = 100 time steps). **f** Simulated domain-averaged normalized anisotropic stress over time for division-active (*α* > 0) and division-inhibited (*α* = 0) conditions. *N* = 3 ; curves represent mean values. **g** Experimental measurements of the average anisotropic stress within the whole tissue, for MDCK WT and for mitomycin-C treated MDCK cells. *n* = 24 movies from *N* = 5 independent experiments (WT) ; *n* = 15 movies from *N* = 3 independent experiments (Mito-C). [Scale bar 25 μm (**b**). All error bars show the confidence interval at 95%.].

Time-resolved stress maps confirmed this: cytokinesis was consistently followed by a local drop in anisotropy, whether cells divided under tension or compression (Fig. 4a). Both maximum and minimum principal stresses relaxed in a coordinated way (Fig. S10a), showing that divisions actively dissipate anisotropy along the division axis rather than passively reflecting it. Notably, we observed that when cells divided under tension, the compressive stress generated during cytokinesis counteracted the anisotropic tensile stress (Fig. 4b, c, [+] distribution). Alternatively, under compression, while the initial load acted perpendicular to the cell axis, cytokinesis generated compression along the division axis, thereby also reducing the difference between principal stresses (Fig. S10b–d) and thus decreasing stress anisotropy, despite increasing the local compression (Fig. 4b, c, [-] distribution).

Simulations with a minimal active gel model^47,48^ captured this prediction: inserting artificial divisions at high-anisotropy sites triggered local stress relaxation (Fig. 4d, e and Fig. S11), while tissues without divisions remained mechanically frozen (Fig. 4f).

To test whether divisions are required for stress relaxation, we blocked proliferation using mitomycin-C. In these arrested monolayers, anisotropic stress no longer declined with time but instead accumulated and remained elevated (Fig. 4g). Moreover, by inhibiting proliferation at different stages of tissue densification, we were able to preserve distinct magnitudes of anisotropic stress corresponding to the level reached at the time of inhibition (Fig. S12a–c). This result rules out passive remodeling as the main driver of stress dissipation: without divisions, the feedback loop is broken and anisotropy persists. Thus, cytokinesis is not merely correlated with stress relaxation but is necessary for maintaining stress homeostasis.

To further investigate the role of cytokinesis, we perturbed cytokinetic contractility using increasing doses of blebbistatin (Fig. S12d–f). Consistent with our hypothesis, inhibition of contractile ring constriction impaired stress relaxation and led to the persistence of anisotropic stress, supporting a direct role for cytokinesis in the dissipation of mechanical anisotropy. Conversely, drug washout restored the feedback loop, enabling the monolayer to progressively relax accumulated stress as cell division resumed (Fig. S12g–i).

**Fig. 5:**
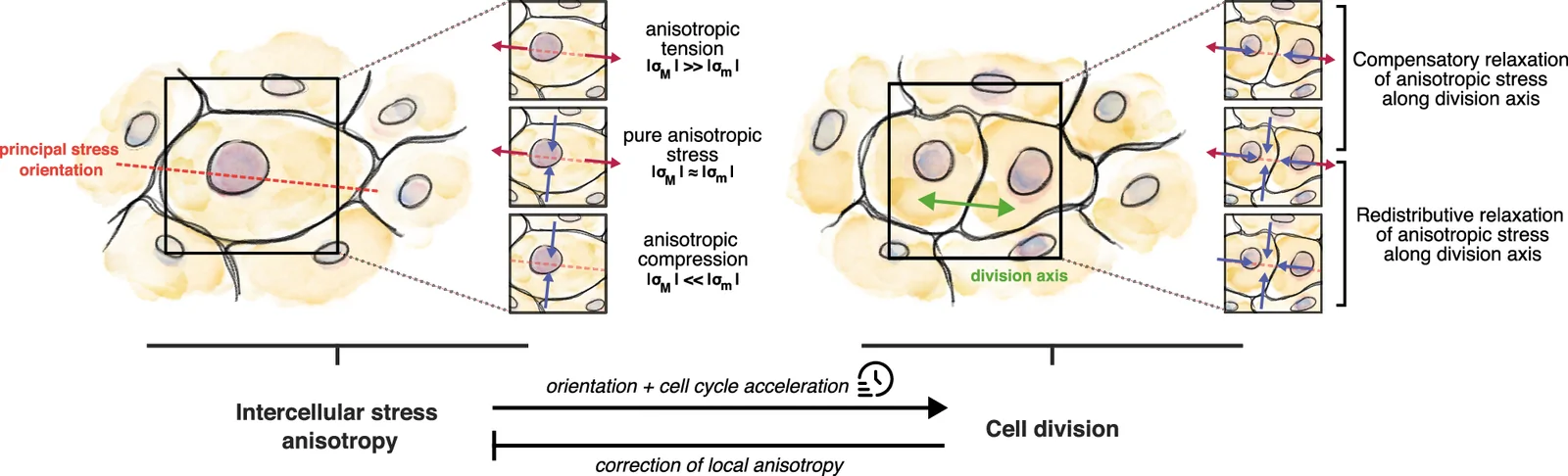
A principal-stress rule for cell division in epithelia. Cartoon summarizing the proposed mechanism : negative feed-back loop between anisotropic stress levels and cell proliferation in epithelia, across various coupling situation of principal stresses.

Together, these findings define a negative feedback loop: anisotropy promotes division, and division releases anisotropy (Fig. 5). This reciprocal coupling functions as a mechanical statos during growth: a feedback system in which stress anisotropy both drives and is dissipated by division, maintaining tissues in a dynamically stable state. Remarkably, this mechanism appears general and persists across different epithelial cell types (Fig. S13, Movie S6-7).

## Discussion

Taken together, these results identify a general physical principle governing how a dense active material relieves internal mechanical stress: through a discrete structural remodeling event, cell division, oriented along the axis of maximal stress anisotropy. When viewed alongside the literature (Supplementary Table 1), this principal-stress rule is not a special case but a genuine extension of the classical long-axis rule: in crowded tissues, where cell elongation and actin-based orientational order collapse, the orientation of structural remodeling is set not by geometry but by the direction of maximal stress anisotropy. This is consistent with previous reports that anisotropic tension can orient cell division^4,13,15,28,38,49^ even when it mismatches cell shape^14,19,20^, and extends further to cases governed by anisotropic compression^6,16,17,50,51^ or an unresolved isotropic stress state^3,4^. That it remains predictive in vitro and ex vivo, across densities, cell types, and mechanical states, suggests this is a material property of dense epithelia, not a peculiarity of a single system.

Beyond orientation, anisotropy also regulates when cells divide by shortening S-G2-M, while cytokinesis dissipates anisotropy, forming a negative feedback that stabilizes the tissue’s mechanical state. Thus, division is both driven by and acts upon the stress landscape, providing a unified mechanical feedback loop for epithelial homeostasis (Fig. 5). Such coupling may represent a general strategy by which dense active materials avoid runaway internal stress while continuing to grow.

This loop provides a mechanical statos: proliferation is triggered by, and simultaneously relieves, stress anisotropy. Mitomycin-C and blebbistatin experiments confirmed the causal role of division in this balance, showing that when divisions were blocked or impaired, anisotropic stress remained elevated rather than relaxing. Such coupling may underlie how epithelia maintain growth while preventing mechanical overload.

Beyond this global homeostat, the stress field also contains finer-grained structures in the form of topological defects. Unlike shape- or actin-based defects, which vanish in crowded tissues, stress defects persist and correlate with division patterns: cells avoid dividing at defect cores but proliferate more frequently in surrounding regions of high anisotropy. These results highlight defects as potential organizing centers for division, opening the door to future studies of how stress defects interact with flows, tissue remodeling, and possibly even the defect dynamics familiar from active matter physics.

This principle has implications that extend beyond epithelial biology to the general mechanics of dense active and disordered materials. During development, anisotropic stress provides a robust orientational signal when geometric cues fluctuate, driving oriented remodeling that shapes tissue architecture^28,43,52^. In wound healing, stress anisotropy concentrated at wound edges^33,53^ may explain reoriented divisions that promote closure. In cancer, failure to relieve accumulated anisotropy may permit unchecked structural remodeling and invasive growth^45,54^. And in engineered tissues, organoids, and synthetic active materials, deliberately imposed stress anisotropy could provide a route to program the geometry of structural remodeling, offering a design principle for materials that self-organize under internal mechanical control^42^. By shifting the organizing variable from geometry to stress, this work establishes a constitutive-like, testable rule for how dense active materials restructure under internal mechanical load, linking stress, structural remodeling, and homeostasis within a single quantitative framework.

## Methods

### Cell lines culture

The following cell lines were used:

- MDCK-II (ATCC CCL-34)
- MDCK-II CAAX GFP (recently described in^55^)
- MDCK-II FUCCI cell cycle reporter (described in^52^)
- MCF10A (ATCC CRL-10317)
- N/TERT1 keratinocytes (gift from the Niessen laboratory (CECAD Cologne, Germany, generated by the Rheinwald laboratory, Harvard), first described in^56^).

MDCK-II WT, MDCK-II FUCCI and MDCK-II CAAX GFP cells were cultured in DMEM (GlutaMAX, supplemented with high glucose and pyruvate, Life Technologies), with 10% fetal bovine serum (FBS, Life Technologies) and 1% penicillin-streptomycin (Life Technologies) at 37 °C with 5%. MCF-10A cells were grown in DMEM-F12 (Gibco) containing 10% penicillin-glutamine, 10 μg/mL human insulin (Sigma-Aldrich), 100 ng/mL cholera toxin (Sigma-Aldrich), 0.5 mg/mL hydrocortisone (Sigma-Aldrich), 5% horse serum, and 20 ng/ml EGF (PeproTeck) at 37 °C with 5%. Keratinocytes were maintained in growth medium (CnT-Prime, CellnTech, Switzerland) at 37 °C with 5%, and switched to differentiation medium (supplemented with high calcium and growth factors, CnT-Prime-3D, CellnTech, Switzerland) 2 days before imaging. Cell populations were split using 0. 05% trypsin (Merck) twice a week. Before processing, the culture medium was aspirated and cells were rinsed with PBS to remove dead cells and debris. Mycoplasma contamination in cell cultures was routinely checked using a PCR test.

### Organoid preparation and culture

Wild-type C57/Bl6 6–12 weeks-old male mice were provided by the animal house facility of the Institut Jacques Monod, and were used for organoid generation. Following euthanasia by cervical dislocation, the small intestine was collected, rinsed with PBS to remove luminal contents, and opened longitudinally. The tissue was then cut into 3–5 mm fragments and washed again in PBS. Organoid preparation and culture was done exactly following the method described in^57,58^.

H2B-mCherry organoids were generated from mice provided by Renata Basto (Institut Curie, Paris)^59^. VillinCreERT2-tdTomato organoids were generated from mice provided by Danijela Vignjevic (Insitut Curie, Paris)^60^.

### Sample preparation

For TFM experiments, cells were grown for 15–20 hours until they formed a monolayer at 90–100% confluency. Prior to imaging, the culture medium was aspirated, and cells were rinsed with PBS to remove dead cells and debris. For TFM experiments involving inhibition of cell division, cells that reached 95% confluency were incubated with mitomycin-C (Sigma-Aldrich) at a concentration of 20 μM for 45 minutes. The medium was then removed, cells were rinsed with PBS, and incubation continued for 12–15 hours to allow all cells to reach the mitotic arrest stage induced by mitomycin-C, prior to imaging. For experiments at different stages of densification, the same protocol was applied, but mitomycin was simply added X days after reaching 95% confluency (X being equal to either 1, 2 or 3). For culture of organoids in 2D, 3D organoids were cultured in conditioned media for at least 3 days before use, mechanically broken and used to generated 2D monolayers following the method used in^35,58^. The organoid-derived monolayers were cultured for 10 days prior live imaging. For TFM experiments involving inhibition of myosin-II, cells that reached 100% confluency were incubated with blebbistatin (Sigma-Aldrich), at either 10, 20, or 50 μM. Control were realized with a corresponding volume of 50 μM DMSO.

For fixed staining, cells that reached the desired density were first rinsed three times with PBS, then incubated with 4% paraformaldehyde (PFA) for 20 minutes at room temperature. Cells were subsequently permeabilized with 0.5% Triton X-100 for 20 minutes. After three 5-minute washes in PBS, samples were blocked with 1% bovine serum albumin (BSA) and 10% fetal bovine serum (FBS) in PBS for 1 hour at room temperature. Samples were then washed three times in PBS, and the actin cytoskeleton was stained using either phalloidin-Alexa Fluor 488 (Invitrogen) or phalloidin-Alexa Fluor 568 (Life Technologies), diluted at 0.5% in PBS. Nuclei were stained with Hoechst (Thermo Fisher), diluted at 0.05% in PBS, for 3 hours at room temperature. Samples were stored at 4 °C until imaging.

### Traction force protocol

Soft biocompatible polymer coated substrates for TFM were prepared following^61^. Briefly, CY52-276A and CY52-276B polydimethylsiloxane (PDMS, Dowsil, Dow) were mixed using different weight ratios (1:1 to achieve 15 kPa, 5:6 to achieve 30kPa) and then poured into microfabrication polystyrene petri dishes (Bio-One, Greiner). The prepared surfaces were spin-coated for 30 seconds at 500 rpm in order to obtain a layer of thickness around 100 μm and then cured at 80 °C for 2 hours. Once cured, each dish was silanized using a 10% APTES ((3-aminopropyl)triethoxysilane, Sigma-Aldrich) solution diluted in absolute ethanol for 15 min and then washed with absolute ethanol 3 times before being dried at 80 °C for 10 min. 200 nm Cy3 red carboxylated fluorescent beads (FluoSpheres, Invitrogen) were diluted in water with a ratio of 2:1000 and disaggregated using an ultrasonic bath for 10 min. The beads solution was filtered using a 0.45 μm filter and incubated on TFM substrates for 15 min, protected from light. The dishes were washed with water 3 times and dried at 80 °C for 3 minutes, and proper homogeneous deposition was checked under microscope. Prior to cell seeding, prepared substrates were coated with 50 μg/mL fibronectin (Sigma) for 45 min and washed 3 times with PBS. Cells were imaged for 24h and beads resting positions were obtained at the end of each experiment by adding 200 μL of 10% sodium dodecyl sulfate (SDS) in the medium in order to remove the cells.

For organoids experiments, 5 kPa soft polyacrylamide (PAA) were used and prepared according to^57^. Briefly, glass coverslips were cleaned in an ethanol bath, sonicated for 1 min, dried at 80 °C for 15 min, and then exposed to plasma cleaning for 10 min. Afterward, the coverslips were incubated for 30 min in a silane solution containing 2% 3-(trimethoxysilyl)propylmethacrylate (Sigma-Aldrich, no. 440159) and 1% acetic acid in ethanol. The silanized coverslips were rinsed with ethanol, dried at 80 °C for 1 h, and stored at room temperature. A freshly prepared polyacrylamide gel mixture (acrylamide, bis-acrylamide, ammonium persulphate, and TEMED in PBS, proportions as described in^57^) containing 4% fluorescent beads (FluoSpheres, Invitrogen) was cast onto the coverslips and allowed to polymerize at room temperature for 1h to form a 100 μm-thick gel. The resulting substrates were stored in PBS at 4 °C. Before use, PAA gels were functionalized with Sulpho-SANPAH (Cultek), activated under 365-nm UV light for 10 min, and coated with a solution of rat-tail type I collagen (First Link UK) and laminin-1 (Sigma-Aldrich) in PBS, followed by overnight incubation at 4 °C, as described in^35^.

### Microscopy and data visualization

Time-lapse traction force microscopy (TFM) was performed using a live-cell epifluorescence microscope (BioStation IM-Q, Nikon or Nikon confocal CSU), equipped with either a 10× or 20× phase-contrast air objective and a standard incubation chamber. Cells were maintained at 37 °C with 5%. The monolayer was imaged using either phase contrast or DIC, and fluorescent beads were visualized using epifluorescence. For cell shape characterization, MDCK CAAX-GFP cells were used and imaged using epifluorescence. Time-lapse videos were recorded at multiple positions every 5 minutes (MDCK) or every 10 minutes (MCF10A and keratinocytes), for a typical duration of approximately 24 hours.

Confocal microscopy was performed using a laser scanning confocal microscope (ZEISS LSM 980), equipped with a 40× or 63× oil-immersion objective and an Airyscan 2 module. MDCK WT cells were grown, fixed and mounted on a glass-bottom imaging dish upon reaching the desired cell density. Unless otherwise specified, all images or Z-stacks were acquired in Airyscan mode without signal averaging, and automated deconvolution was performed using the microscope software (ZEISS ZEN Blue).

Most images were visualized using ImageJ^62^, with brightness and contrast adjusted as needed. For Z-stacks, maximum intensity projections were generated. In cases where physical parameters such as the nematic director or principal stress orientation were overlaid, images were visualized using MATLAB. Brightness and contrast adjustments were also performed in MATLAB using the Image Processing Toolbox.

### Analysis method

Unless stated otherwise, all codes for the following experimental analysis were written in MATLAB using built-in functions, standard toolboxes, or previously published tools implemented in MATLAB.

#### Shape characterization

Geometrical characterization of the cells during monolayer packing was performed using from MDCK CAAX-GFP cells signal. Cells were segmented using Cellpose with the pretrained model cyto3^63^. Customized binary images of the segmented cells were generated (see Fig. S4a), and a filter was applied based on the average area of each object to discard falsely detected cells, using the regionprops function of MATLAB. Cells with an area smaller than 80 μm^2^ were excluded. The area, perimeter, aspect ratio (via ellipse fitting), and center-of-mass position were computed for each remaining object, post filtering. Using the formula ${\rho }_{0}=\frac{P}{\sqrt{A}}$, where *P* is the perimeter and *A* is the area of the cell, the cell shape index *ρ*_0_ was also computed. The center-of-mass positions were used to calculate the average distance between neighboring cells using a Euclidean metric.

#### Collective cell dynamics

Dynamical characterization during monolayer densification was performed by tracking individual cell movements within the monolayer. Cell tracking was conducted using TrackMate^64^ on brightfield time-lapse images, employing a LoG detector combined with a median filter for cell detection, followed by an advanced Kalman tracker for tracking. The initial search radius was set to 15 μm and allowed to increase up to 20 μm. Tracks were permitted to have one undetected frame. Each track provided the time-dependent center-of-mass position ***r***_*i*_(*t*) = [*x*_*i*_(*t*), *y*_*i*_(*t*)] for a given object *i*, enabling computation of the mean square displacement (MSD) as:

$$MSD(\Delta t)=\langle \parallel {{{{\boldsymbol{r}}}}}_{i}(t+\Delta t)-{{{{\boldsymbol{r}}}}}_{i}(t){\parallel }^{2} \rangle$$

where the average $\langle \cdot \rangle$ is taken over all cells *i* and all time points *t*. Tracks were grouped into five time windows of 5 hours each (0-5 h, 5-10 h, etc.), and the MSD was computed over each period. The global cellular velocity field was quantified using the Particle Image Velocimetry toolbox PIVlab^65^. Displacement field was computed using an interrogation window of 64 × 64 pixels with 50% overlap. The resulting vector field was first smoothed using a second interrogation window of 32 × 32 pixels, and then using the built-in averaging statistical algorithm in the plugin. Velocity field was then computed accordingly, knowing the duration between two consecutive frames.

#### Nematic analysis

The nematic director field was approximated using intensity gradients within each cell, excluding cell edges. To compute the intensity gradient, the OrientationJ plugin^66^ in ImageJ was used. The resulting field was coarse-grained using a moving square window of 100 pixels with 64 pixels of overlap. For visualization, the field was interpolated so that each director approximately corresponds to one cell.

Nematic topological defects were detected using a winding number approach, following a previously established pipeline^26,67,68^. For each detected $+\frac{1}{2}$ or $-\frac{1}{2}$ topological defect, the local divergence of the nematic director field was used to determine the orientation of the defect, as proposed in^69^.

To quantify the loss of nematic ordering as cells lose their elongated shape as cell density increased, we defined an “apparent" scalar order parameter *q*_*a*_ (Fig. S4c) as ${q}_{a}= < \nu ({{{\boldsymbol{r}}}})\,q({{{\boldsymbol{r}}}}) >$ where *q*(***r***) is the local classical nematic scalar order parameter at the position ***r***, computed using a local window *w* such as:

$$q({{{\boldsymbol{r}}}})=2{\cos }^{2}({\theta }_{{{{\boldsymbol{r}}}}}-{\theta }_{w})-1$$

Here, *θ*_***r***_ is the local director orientation at the position ***r***, and *θ*_*w*_ is the average director field orientation within the window *w*. In our experiments, *w* is a square of size 50 μm. The function *ν*(***r***) acts as a pseudo-flag based weight using the local shape index *ρ*_0_(***r***), and is defined as:

$$\nu ({{{\boldsymbol{r}}}})=\left\{\begin{array}{ll}1.5\quad &\,{\mbox{if}}\,{\rho }_{0}({{{\boldsymbol{r}}}}) > 4\\ 0\quad &\,{\mbox{if}}\,{\rho }_{0}({{{\boldsymbol{r}}}}) < 3.81\\ 1\quad &\,{\mbox{otherwise}}\end{array}\right.$$

where *ρ*_0_(***r***) is the local shape index of the cell at the position ***r***. Simply put, the scalar order parameter was weighted by the local shape index to reduce its contribution in regions where cells adopt a more hexagonal morphology, thereby capturing the loss of nematic order during tissue densification.

#### Traction forces and stress measurement

Images of bead displacements relative to their relaxed positions were preprocessed using the Image Stabilizer plugin in ImageJ^70^, and background illumination was corrected to reduce noise. The beads displacement field was obtained using PIVlab^65^, with an interrogation window of 32 × 32 pixels and a 50% overlap. The corresponding traction force field was then inferred using Fourier transform traction cytometry, knowing theoretical substrate stiffness (15 or 30 kPa in our experiments) and a regularization parameter of 9 × 10^−9^. From the traction force field, we computed the 2D stress tensor across the tissue using BISM^31^, with a regularization parameter of *Λ* = 10^−6^. The 2D stress tensor was then diagonalized in order to compute the principal stress direction as well as the maximum and the minimum principal stress, *σ*_*M*_ and *σ*_*m*_ (see Supporting Information).

Because principal stress orientation can be mathematically computed even in purely isotropic regions where principal stress orientation lacks physical meaning, we defined an apparent scalar order parameter for the stress tensor, $q^{\prime}$, similarly to the nematic case. Here, the weighting factor is defined as $\nu ^{\prime}=\sqrt{3}\frac{{\sigma }_{aniso}}{{\sigma }_{VM}}$ where *σ*_*VM*_ is the von Mises stress such as ${\sigma }_{VM}=\sqrt{{\sigma }_{M}^{2}+{\sigma }_{m}^{2}-{\sigma }_{M}{\sigma }_{m}}$. Defined that way, this anisotropic ratio (Fig. S9d-e) is bounded between 0 (when *σ*_*M*_ = *σ*_*m*_) and 1 (when *σ*_*M*_ = − *σ*_*m*_). The apparent stress scalar order parameter $q^{\prime}$ was then defined as $q^{\prime}= < \nu ^{\prime} ({{{\boldsymbol{r}}}})\,q^{\prime} ({{{\boldsymbol{r}}}}) >$ with $q^{\prime} ({{{\boldsymbol{r}}}})$ the local scalar order parameter computed using the director of the principal stress directions.

To detect stress topological defects, we used the same pipeline described above for nematic topological defects. To track the movement of stress defects over time, we generated movies in which the positions of each defect were represented as white objects on a black background. These binary position images were then analyzed using TrackMate, and all parameters used to characterize the motion of each trajectory were retrieved from the TrackMate analysis. Defect lifetime was defined as the duration of the track. The net displacement was computed as *d*_*net*_ = ∣∣***r***_***f***_ - ***r***_***0***_∣∣ where ***r***_***f***_ and ***r***_***0***_ are the final and initial positions of the considered defect. The confinement ratio was defined as $CR=\frac{{d}_{net}}{{d}_{tot}}$ with *d*_*tot*_ being the total cumulated distance traveled by the considered defect, and *d*_*net*_ the actual net distance traveled by the considered defect. A confinement ratio approaching zero indicates restricted (caged) displacement, whereas a ratio approaching one corresponds to a straight-line trajectory. A binary spatial map of all cumulative defect positions (independent of topological charge) was then generated for each video, and these maps were subsequently used to compute the average spatial distribution of distances from defect cores. When comparing defects positions to random positions, 100 random positions were taken for each frame.

#### Micropatterning and laser ablation

Microfabrication was done following^26,61,71^. Mold of ellipse dimensioned as 150 x 300 μm were obtained using standard lithography methods. PDMS (SYLGARD 184, Dow Corning) was prepared by mixing the base with a curing agent at a ratio of 1:10, poured over the mold, degassed and then cured at 80 °C for 2 h. Stamps were peeled of the mold and stored, protected from light and humidity. When utilized, a fibronectin solution (50 μg/mL, Sigma) was incubated covering the surface of the mold for 45 min. The surface was cleaned using a gentle air flow, and the stamps were gently pressed against the bottom of a PDMS substrate for about 1 min. Patterns were then incubated with a solution of 2% Pluronic F-127 (Sigma) for 1 h to passivate the areas outside the patterns.

MDCK CAAX GFP cells were seeded on PDMS patterned dish and grown to confluency inside each ellipse. Before wound induction, dishes were rinsed using warm PBS and provided with a fresh culture medium. Laser ablation was done using a spinning-disk CSU-X1 with a fluorescence recovery after photo bleaching module (Yokogawa) and a  × 40/1.2 water immersion objective. Briefly, ellipse were ablated along their central short axis by focusing an ultraviolet laser (355 nm, pulse duration 3-5 ns, laser power 450 mW) for 1 s. Each sample was imaged during 5 s before ablation and until 1 min after ablation, using 1s intervals. The recoil velocity was measured by manually segmenting the wound separation in the direction of the ellipse long axis over time and plotting the corresponding size rate evolution.

#### Cell division events

For MDCK WT, N/TERT1 keratinocytes, MCF10A and organoids data, position and orientation of cytokinesis events were manually detected in a homogeneous manner, without prior knowledge of the mechanical state of the tissue. To ensure a spatially uniform distribution of marked events, each image was divided into four quadrants, and an equal number of division events were marked in each. The typical number of divisions annotated per position in a given experiment was 125 for MDCK, 80 for the model systems. When a parameter was analyzed around a cell division event, it was generally evaluated locally at the position of the event. In Fig. 4 and Supplementary Fig. S10, however, the stresses were computed as the median within a 50 μm square window centered on the event to provide a quantitative measure representative of the local stress patterns displayed in these figures.

For MDCK FUCCI data, binary masks for each signal (G1/G0 and S/G2/M) were generated after preprocessing using MATLAB’s Image Processing Toolbox. On the figures, the nuclear signal is pink when the cell is in G1 or G2 phase, and green when being in S, G2 or M phase. Specifically, the imbinarize function was applied, followed by morphological operations such as erosion and dilation. Objects too small to represent nuclei were filtered based on area thresholds. Only complete trajectories, corresponding to cells entering and exiting a given FUCCI state within the imaging interval, were retained for analysis. Each binary image was analyzed using TrackMate to determine the lifetime of each detected object, corresponding by construction to the duration of a particular cell cycle phase. A two-dimensional spatial map of each phase duration was then constructed by assigning to each position **r** the average duration of all tracked cells observed at that location throughout the experiment. Formally, for a given spatial map of a considered cell cycle phase duration ${D}_{phase}^{\tau }$, ${D}_{phase}^{\tau }$ is computed as ${D}_{phase}^{\tau }({{{\boldsymbol{r}}}})=\langle {\tau }_{phase}({{{\boldsymbol{r}}}}) \rangle$ where $\langle \cdot \rangle$ denotes the average over cell cycle duration *τ*_*phase*_ of the considered cell cycle phase at the position ***r*** during the course of experiment. A similar approach was adopted to characterize the average isotropic mechanical state experienced by cells over the course of the experiment. The isotropic stress field was first averaged over time, yielding a two-dimensional map of the mean isotropic stress. This map was subsequently discretized into three categories (tensile regions, corresponding to averaged isotropic stresses larger than 100Pa, compressive regions, corresponding to averaged isotropic stresses smaller than  − 100Pa, and mixed regions, corresponding to intermediate values) and compared with the generated cell cycle S-G2-M duration map.

### Plotting, statistics and reproducibility

All plotted heatmaps were smoothed using linear interpolation. When plotting an orientation field, magnitude of the elongation is weighted by the anisotropic aspect ratio (for stress orientation) or by the shape aspect ratio (for nematic director). All plots and graphs display the median of the corresponding distribution, unless stated otherwise. Similarly, all error bars represent 95% confidence intervals, unless otherwise specified. Statistical tests were conducted using MATLAB’s Statistics and Machine Learning Toolbox, and *p* values were reported up to four decimal places to take into account the significance. Probability Density Function (PDF) statistical distributions of any parameters were plotted using MATLAB’s Statistics and Machine Learning Toolbox, using the function histogram2 for 2D distribution, and histcounts for 1D distribution, with the normalization parameter set on pdf. All selected images correspond to quantified parameters and depict a representative state of the independent experiments reported. Unless stated otherwise, all images are representative of at least *N* = 2 independent experiments. Sketches of proposed mechanisms and illustrations were created by hands as digital drawings using Clip Studio Paint and Inkscape.

### Lyotropic active nematic model with tunable cell concentration

We developed a minimal continuum model that describes epithelia as a lyotropic active gel with tunable cell concentration (*ϕ*)^47,48^. The system is characterized by three key fields: the cell concentration *ϕ*, the velocity field **u**, and the nematic order parameter **Q** = 2*q*(**nn** − **I**/2), where **n** is the nematic director and *q* is the magnitude of nematic order. The concentration *ϕ* varies from 0 (low density) to 1 (high density), representing local cell density.

The evolution of the nematic tensor is governed by

1 $$({\partial }_{t}+{{{\bf{u}}}}\cdot \nabla ){{{\bf{Q}}}}-{{{\bf{S}}}}={\Gamma }_{Q}{{{\bf{H}}}},$$

where **S** = *λ***E** − (***ω*** ⋅ **Q** − **Q** ⋅ ***ω***) is a generalized advection term, with **E** = ( ∇ **u** + ∇ **u**^*T*^)/2 the strain rate tensor and ***ω*** = ( ∇ **u**^*T*^ − ∇ **u**)/2 the vorticity tensor. The alignment parameter *λ* depends on particle shape: *λ* > 0 for rod-like particles, *λ* < 0 for disk-like particles, and *λ* = 0 for spherical particles. The molecular field **H** is defined as ${{{\bf{H}}}}=-\frac{\partial {{{{\mathcal{F}}}}}_{{{{\rm{LC}}}}}}{\partial {{{\bf{Q}}}}}+\nabla \cdot \left(\frac{\partial {{{{\mathcal{F}}}}}_{{{{\rm{LC}}}}}}{\partial (\nabla {{{\bf{Q}}}})}\right)$, where ${{{{\mathcal{F}}}}}_{{{{\rm{LC}}}}}$ represents the nematic elastic free energy based on the Oseen-Frank form under the single elastic constant approximation^72^: ${{{{\mathcal{F}}}}}_{{{{\rm{LC}}}}}=\frac{1}{2}K{(\nabla {{{\bf{Q}}}})}^{2}$.

Cell division is incorporated as a local source term in the equation for *ϕ*. This formulation has been widely used to describe collective cell dynamics and reproduce experimentally observed proliferation and stress patterns^73,74^. The dynamics of *ϕ* obey:

2 $${\partial }_{t}\phi+\nabla \cdot ({{{\bf{u}}}}\phi )={\Gamma }_{\phi }{\nabla }^{2}\mu+\alpha \phi,$$

where *Γ*_*ϕ*_ is the mobility, *α* is the division rate, and the chemical potential is given by

3 $$\mu=\frac{\partial {{{\mathcal{F}}}}}{\partial \phi }-\nabla \cdot \left(\frac{\partial {{{\mathcal{F}}}}}{\partial \nabla \phi }\right).$$

The total free energy density ${{{\mathcal{F}}}}$ consists of both nematic and Ginzburg-Landau contributions:

4 $${{{\mathcal{F}}}}={{{{\mathcal{F}}}}}_{{{{\rm{LC}}}}}+{{{{\mathcal{F}}}}}_{{{{\rm{GL}}}}},$$

where

5 $${{{{\mathcal{F}}}}}_{{{{\rm{GL}}}}}=\frac{{A}_{\phi }}{2}{\phi }^{2}{(1-\phi )}^{2}+\frac{{K}_{\phi }}{2}{(\nabla \phi )}^{2},$$

describes phase separation and interfacial tension between isotropic and nematic regions^75^.

The velocity field **u** evolves according to the momentum conservation equation:

6 $$\rho ({\partial }_{t}+{{{\bf{u}}}}\cdot \nabla ){{{\bf{u}}}}=\nabla \cdot {{{\mathbf{\Pi }}}},$$

where *ρ* is the effective mass density and **Π** is the total stress tensor, composed of active, viscous, elastic, and capillary contributions. The active stress, capturing contractility-driven nematic interactions, is modeled as:

7 $${{{\mathbf{\Pi }}}}^{{{{\rm{active}}}}}=\zeta (1-\phi ){{{\bf{Q}}}},$$

where *ζ* is the activity coefficient. This form reflects the experimentally observed suppression of activity with increasing cell density (Fig. S3C,D). The viscous stress is given by:

8 $${{{\mathbf{\Pi }}}}^{{{{\rm{viscous}}}}}=2\eta {{{\bf{E}}}},$$

with *η* the shear viscosity. The elastic stress is expressed as:

9 $${{{\mathbf{\Pi }}}}^{{{{\rm{elastic}}}}}=-P{{{\bf{I}}}}-\lambda {{{\bf{H}}}}+{{{\bf{Q}}}}\cdot {{{\bf{H}}}}-{{{\bf{H}}}}\cdot {{{\bf{Q}}}}-\nabla {{{\bf{Q}}}}:\frac{\partial {{{\mathcal{F}}}}}{\partial \nabla {{{\bf{Q}}}}},$$

where *P* is the reference pressure, set to zero in this study. The capillary stress accounts for chemical potential gradients and interfacial effects:

10 $${{{\mathbf{\Pi }}}}^{{{{\rm{cap}}}}}=({{{\mathcal{F}}}}-\mu \phi ){{{\bf{I}}}}-\nabla \phi \left(\frac{\partial {{{\mathcal{F}}}}}{\partial \nabla \phi }\right),$$

where the first term represents a bulk pressure arising from crowding-induced increases in intracellular osmotic pressure, as reported in^76^.

We use a hybrid Lattice Boltzmann algorithm to solve the coupled equations of motion. Full details of the numerical implementation are available in previous studies^77,78^. Unless otherwise specified, simulations are performed using the following parameter values (in lattice units): Γ_*Q*_ = 0.3, *λ* = 0.3, *K* = 0.002, Γ_*ϕ*_ = 0.05, *ζ* = 0.02, *A*_*ϕ*_ = 0.03, *K*_*ϕ*_ = 0.06, and *η* = 2/3. For simulations with uniform cell concentration, we set *α* = 0 and gradually increased *ϕ* from 0.3 to 0.7. For simulations with localized division, the initial cell concentration was set to *ϕ* = 0.5, and division events were triggered at sites of high anisotropic stress, defined within a radius of 3 lattice units around the local maximum of anisotropic stress. Division events were introduced every *t*_*d*_ = 150 time steps and persisted for Δ*t* = 100 time steps. The simulation domain was set to 200 × 200 lattice units and run for a total of 20,000 time steps. All results were computed from the final 3000 steps to ensure statistical steady-state.

## Supplementary information

Supplementary Information

Description of Additional Supplementary Files

Movie 1

Movie 3

Movie 2

Movie 4

Movie 5

Movie 6

Movie 7

Transparent Peer Review file

## Data Availability

All data supporting the findings of this study are included within the Article and its Supplementary Information. Source data are provided with this paper. Unprocessed data and raw images are available upon request.

## Code availability

All code used to process the data and to perform the experimental analysis are available upon request. In particular, the code used for performing BISM is available at : ASmushyCell/TFM-BISM-analysis. The simulation code is available at : active-intelligent-matter/mass-cell-division.
