# Supplementary material for: A principal-stress rule for cell division in epithelia
Source: Nat Commun. 2026 Sep 23;17:11062. doi: 10.1038/s41467-026-77813-5 (PMC13691091; doi:10.1038/s41467-026-77813-5)
Supplement: Supplementary file 1 [file 41467_2026_77813_MOESM10_ESM.pdf]

nature portfolio

Peer Review File

A principal-stress rule for cell division in epithelia

Corresponding Author: Professor Benoit Ladoux

This manuscript has been previously reviewed at another journal. This document only contains information relating to versions considered at Nature Communications.

Version 0:

Reviewer comments:

Reviewer #1

(Remarks to the Author)
The revised manuscript is much improved, the authors have made a significant effort to clarify the points I have previously requested, have provided additional analysis, clarified their methods where needed, softened their conclusions where requested to do so provided additional data, analysis detailed justification in the rebuttal letter to further support their work.
I am pleased with the progress effort they showed in improving the manuscript.

Reviewer #2

(Remarks to the Author)
The authors have substantially improved the manuscript by addressing all my concerns. In the previous submission, several experiments results were not explained with sufficient clarity; these issues have now been successfully resolved through more detailed descriptions, improved analysis methods. Experiments (e.g. blebbistatin, mitomycin-C) have been done to respond to some of the reviewers comments. The discussion has also been improved extended. The authors now place their work better within the context of the existing literature (Supp. Table 1), clearly explaining this work's contributions, acknowledging the strength limitations of their findings (e.g. local versus bulk stress, isotropic versus anisotropic stress, area, density, Hertwig's rule).

While there is still some room for further improvements in clarity to enhance readability, the manuscript is well written considerably easier to follow than in its original form.
Overall, this is a rigorous impactful study that makes a relevant contribution to our understanding of how local force asymmetries influence cell division orientation in epithelial tissues. The work will be of considerable interest to researchers working at the interface of physics, biology, mechanobiology.

Reviewer #3

(Remarks to the Author)
I would like to thank the authors for their thorough thoughtful revision of the manuscript for providing a detailed point-by-point response to my comments. I particularly appreciate the substantial effort that the authors have invested in addressing the concerns raised in my previous report. The revised manuscript includes a significant amount of new experimental work, quantitative analysis, simulations, which have considerably strengthened the study addressed the questions concerns raised during the previous round of review.

I have carefully reviewed the revised manuscript the authors' responses find that all of my previous concerns have been satisfactorily addressed. I therefore recommend the current version of the manuscript for publication in Nature Communications.

Open Access This Peer Review File is licensed under a Creative Commons Attribution 4.0 International License, which permits use, sharing, adaptation, distribution and reproduction in any medium or format, as long as you give appropriate credit to the original author(s) and the source, provide a link to the Creative Commons license, and indicate if changes were made.
In cases where reviewers are anonymous, credit should be given to 'Anonymous Referee' and the source.
The images or other third party material in this Peer Review File are included in the article's Creative Commons license, unless indicated otherwise in a credit line to the material. If material is not included in the article's Creative Commons license and your intended use is not permitted by statutory regulation or exceeds the permitted use, you will need to obtain permission directly from the copyright holder.
To view a copy of this license, visit https://creativecommons.org/licenses/by/4.0/
