# Supplementary material for: A principal-stress rule for cell division in epithelia
Source: Nat Commun. 2026 Sep 23;17:11062. doi: 10.1038/s41467-026-77813-5 (PMC13691091; doi:10.1038/s41467-026-77813-5)
Supplement: Supplementary file 2 [file 41467_2026_77813_MOESM1_ESM.pdf]

Supplementary Information for

A principal-stress rule for cell division in epithelia

Authors: Lucas Anger[1], Tianxiang Ma[2], Fanny Wodrascka[1], Andreas Schoenit[1], Kristian Thijssen[2], Satish Kailasam Mani[1], Marc-Antoine Fardin[1], Carine Rosse[1,3], René-Marc Mège[1*], Amin Doostmohammadi[2*], Benoit Ladoux[1,4,5*]

Affiliations:.
[1]Université Paris Cité, CNRS, Institut Jacques Monod, F-75013, France.
[2]Niels Bohr Institute, University of Copenhagen, Copenhagen, Denmark.
[3]Institut Curie, Paris Université Sciences et Lettres, Sorbonne Université, CNRS, UMR144, 75005 Paris, France.
[4]Department of Physics, Friedrich-Alexander-Universität Erlangen-Nürnberg, 91058 Erlangen, Germany.
[5]Max-Planck-Zentrum für Physik und Medizin and Max Planck Institute for the Science of Light, 91058 Erlangen, Germany.

*Corresponding author(s). E-mail(s): rene-marc.mege@ijm.fr; doostmohammadi@nbi.ku.dk; benoit.ladoux@fau.de;

The PDF file includes:

- Supplementary Figures Fig. S1 to S13
- Supplementary Table 1
- Supplementary Notes
- Supplementary References

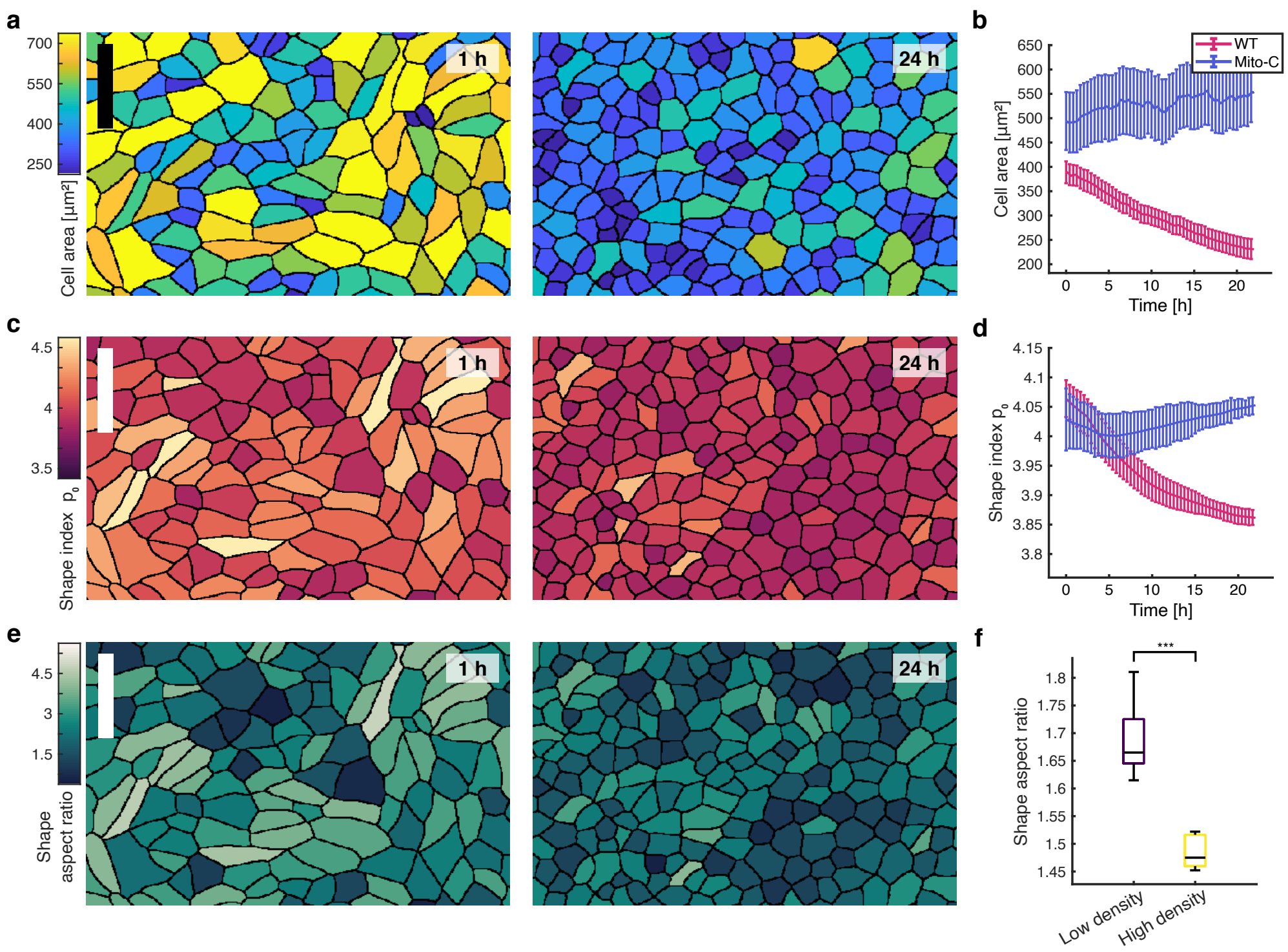

Fig. S1 MDCK monolayer mechanical state evolves with tissue densification. **a**) Left, segmented image of sub-confluent MDCK monolayer at low density, right, same monolayer in high density. Color code is the cell area. **b**) Mean evolution in time of the cell area within the whole tissue, for MDCK WT and for mitomycin-C treated MDCK cells. n = 15 movies from N = 3 independent experiments (WT) ; n = 15 movies from N = 3 independent experiments (Mito-C). **c**) Left, segmented image of sub-confluent MDCK monolayer at low density, right, same monolayer in high density. Color code is the cell shape index. **d**) Mean evolution in time of the cell shape index within the whole tissue, for MDCK WT and for mitomycin-C treated MDCK cells. n = 15 movies from N = 3 independent experiments (WT) ; n = 15 movies from N = 3 independent experiments (Mito-C). **e**) Left, segmented image of sub-confluent MDCK monolayer at low density, right, same monolayer in high density. Color code is the cell aspect ratio. **f**) Average shape aspect ratio for low density (t = 1h) and high density (t = 24h). 45 points, n = 15 movies from N = 3 independent experiments. p = 3.17e-16. [Scale bar 50 µm (a, c, e). P-value from two-sided Mann-Whitney U-test (f).]

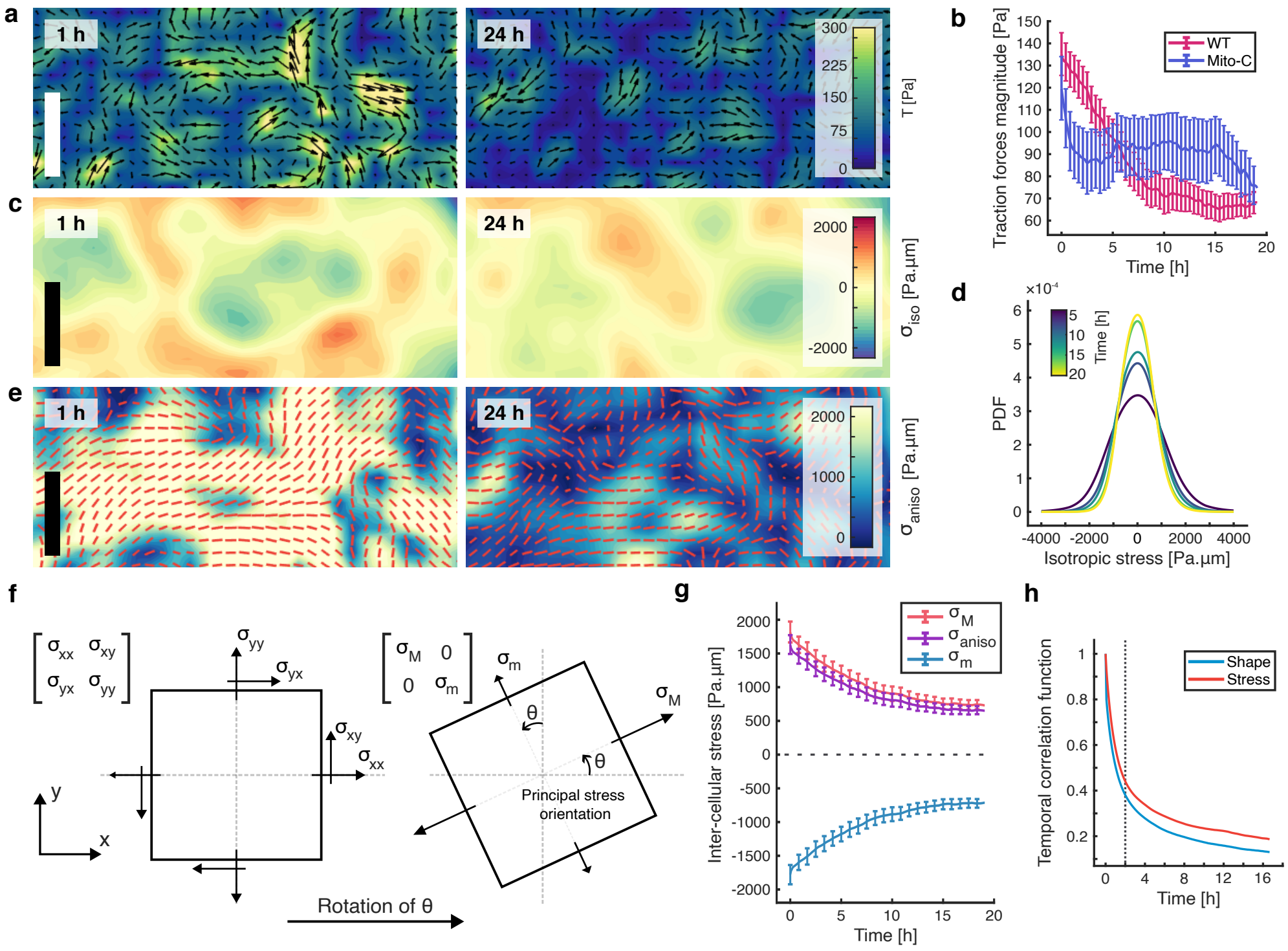

Fig. S2 MDCK monolayer mechanical state is anisotropic in nature. **a**) Left, traction force field of sub-confluent MDCK monolayer at low density, right, same field in high density. Color code is the traction force magnitude. **b**) Average evolution in time of the traction force magnitude within the whole tissue, for MDCK WT and for mitomycin-C treated MDCK cells. n = 24 movies from N = 5 independent experiments (WT) ; n = 15 movies from N = 3 independent experiments (Mito-C). **c**) Left, isotropic stress field of MDCK monolayer in a low density state, right, same field in high density state. Color code is the isotropic stress value. **d**) Probability density function (PDF) evolution of the isotropic stress distribution in MDCK monolayer during monolayer densification. Color code is the time. n = 24 movies from N = 5 independent experiments. **e**) Left, anisotropic stress field of MDCK monolayer in a low density state, right, same field in high density state. Color code is the anisotropic stress magnitude. Principal stress orientation director field in red. **f**) Simplified representation of the transformation allowing the computation of the maximum and minimum stress and defining the principal stress orientation. **g**) Evolution of average inter-cellular stress components in time within the whole tissue (maximum, minimum and anisotropic stress). n = 24 movies from N = 5 independent experiments. **h**) Decay of the temporal correlation function for the principal stress orientation and cell shape orientation. n = 13 positions from N = 3 independent experiments. [Scale bar 70 µm (a, c, e). All error bars show the confidence interval at 95 %.]

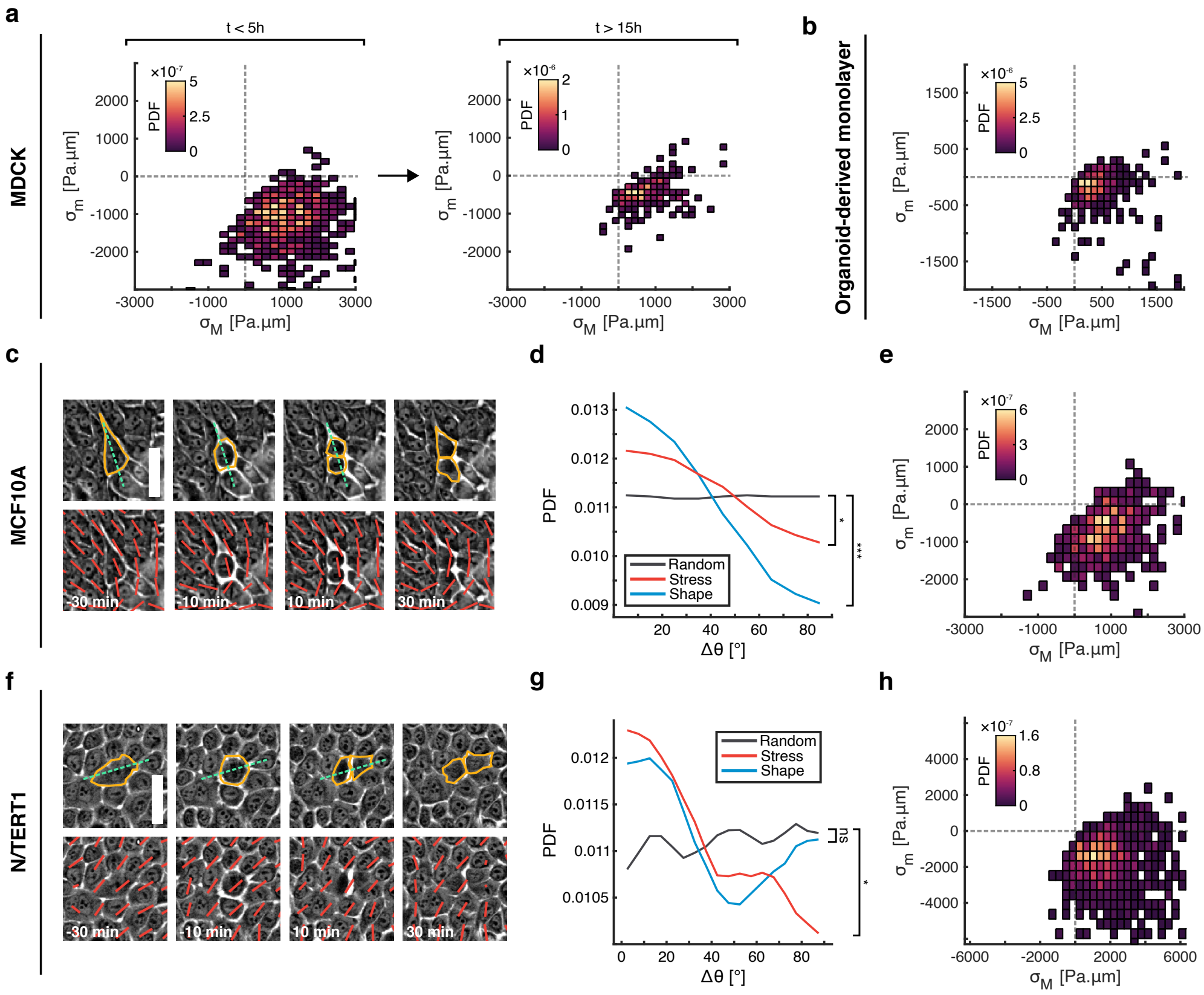

Fig. S3 Principal stress orientation is a stable predictor of cell division orientation in epithelia. **a**) 2D histogram of the maximum and minimum principal stress coupling 30 min before cytokinesis for MDCK monolayer, left, at low density, and right, at high density. Color code is the PDF. 697 cell divisions (($t < 5h$) and 239 cell divisions ($t > 15h$). $n = 13$ movies from $N = 3$ independent experiments. **b**) 2D histogram of the maximum and minimum principal stress coupling 30 min before cytokinesis for organoid-derived monolayer. Color code is the PDF. 620 divisions. $n = 13$ movies from $N = 3$ independent experiments. **c**) Phase contrast image of cell division process, showing in dotted white line cell division orientation, before and after cytokinesis for MCF10A monolayer. Principal stress orientation in red. **d**) Probability distribution function of the angle between cell division orientation and principal stress orientation, for MCF10A monolayer. 354 cell divisions and 2348 random positions. $n = 7$ movies from $N = 2$ independent experiments. $p = 0.0397$ (stress vs random), and $p = 2.25e\text{-}5$ (shape vs random). **e**) 2D histogram of the maximum and minimum principal stress coupling 30 min before cytokinesis for MCF10A monolayer. Color code is the PDF. 354 divisions. $n = 7$ movies from $N = 2$ independent experiments. **f**) Phase contrast image of cell division process, showing in dotted white line cell division orientation, before and after cytokinesis for N/TERT1 monolayer. Principal stress orientation in red. **g**) Probability distribution function of the angle between cell division orientation and principal stress orientation, N/TERT1 monolayer. 850 cell divisions and 2100 random positions. $n = 7$ movies from $N = 3$ independent experiments. $p = 0.04$ (stress vs random), and $p = 0.4311$ (shape vs random). **h**) 2D histogram of the maximum and minimum principal stress coupling 30 min before cytokinesis for N/TERT1 monolayer. Color code is the PDF. 850 divisions. $n = 7$ movies from $N = 3$ independent experiments. [Scale bar 70 µm (c, f). P-value from two-sided Mann-Whitney U-test (d, g)]

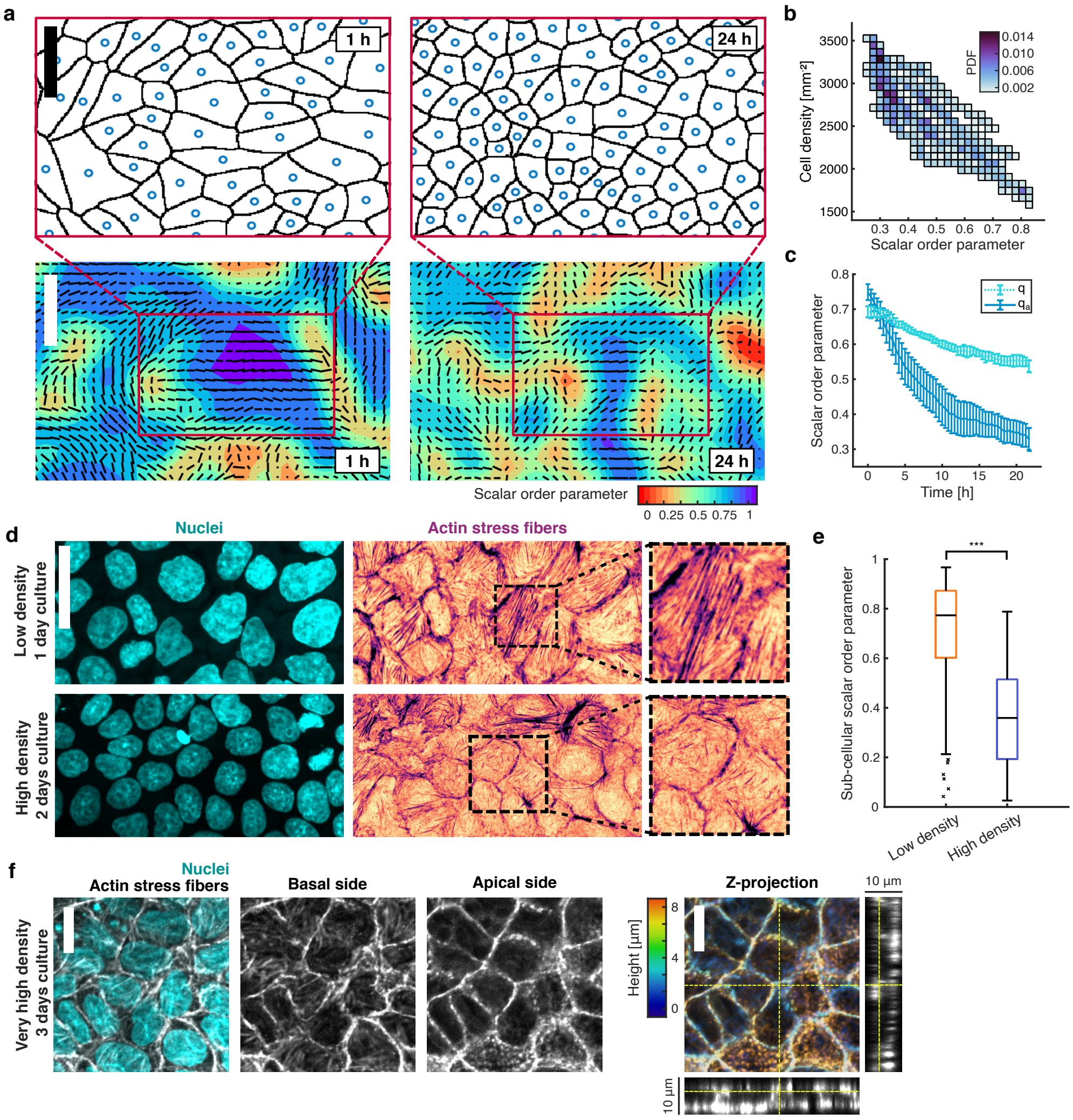

Fig. S4 Tissue densification disrupts nematic order across multiple scales. **a**) Top, left, segmented image of sub-confluent MDCK monolayer at low density, right, same monolayer at high density. Blue dots represent cells center of mass. Bottom, left, scalar local order parameter for the nematic director of MDCK monolayer at low density, right, same monolayer at high density. Color code is the local scalar order parameter. Nematic director field is in black. Magnitude of the elongation is weighed with the cell aspect ratio. The red box correspond to the segmented localization of the top panel. **b**) 2D histogram of the coupling between cell density and scalar order parameter across time. Color code is the probability density function (PDF). n = 15 movies from N = 3 independent experiments. **c**) Average scalar order parameter in time for the nematic director field, using direct (q) computation or normalized (q_a, shape index is used as weights) computation. Using q is not enough to make the order collapse because cell orientations computed past t = 10h do not have a physical meaning anymore (see Materials and Methods). n = 15 movies from N = 3 independent experiments. **d**) Example images of the actin cytoskeleton basal organization (left) for low density monolayer and higher density monolayer state (top and bottom). Right is the corresponding nuclei. **e**) Average intra-cellular actin fiber scalar order parameter for low and high cell density. 75 cells (low), 87 cells (high), n = 14 images (low) and n = 13 images (high) from N = 3 independent experiments. p = 2.24e15. **f**) Typical images of the actin cytoskeleton basal and apical organization at very high cell density, with the corresponding nuclei staining. Left is the corresponding z-projection, colored by the height of each slice. [Scale bar 75 µm (bottom a), 30 µm (top a, d), 10 µm (f). P-value from two-sided Mann-Whitney U-test (e).]

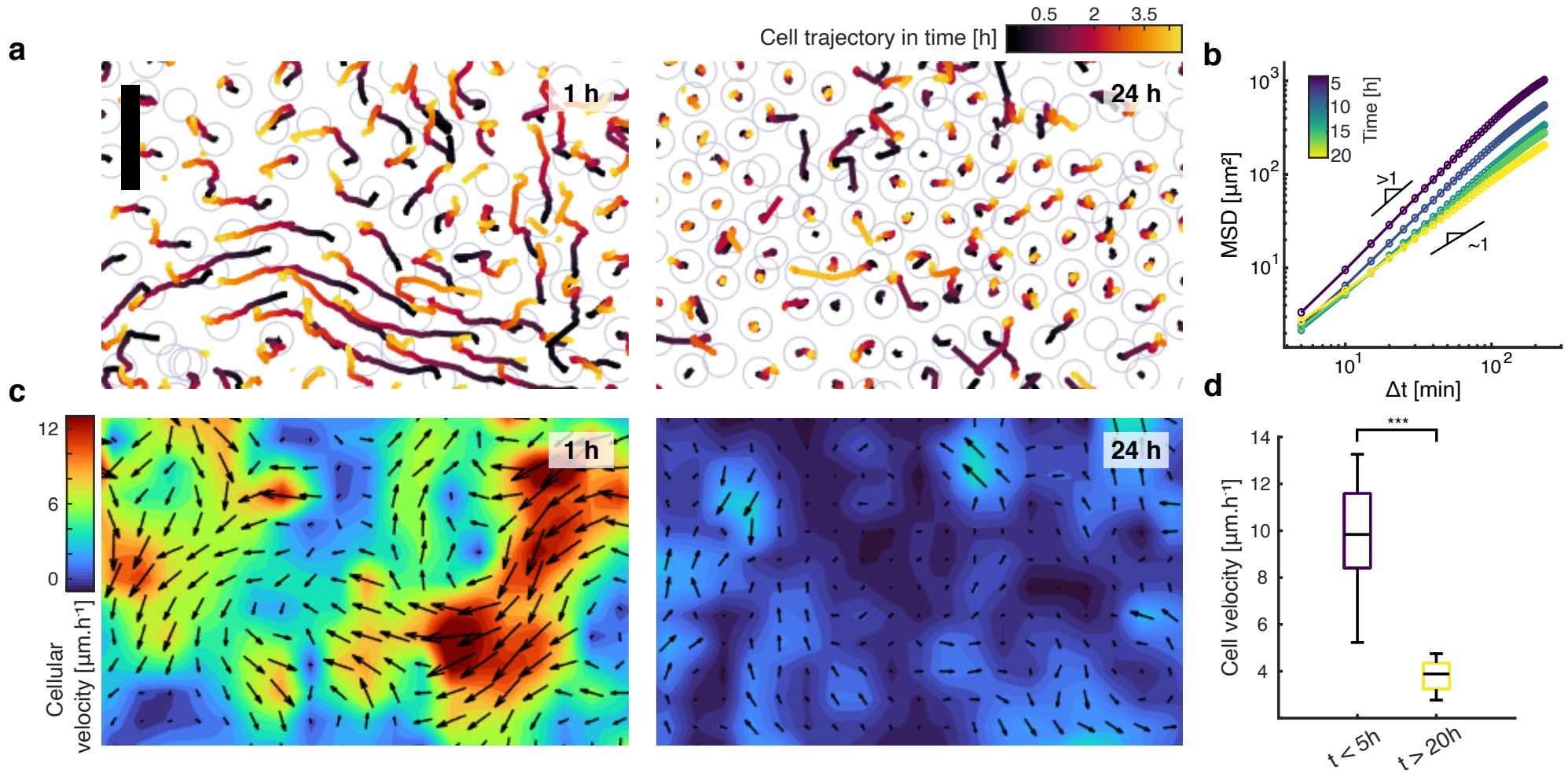

Fig. S5 MDCK cells undergo jamming transition during tissue densification. **a**) Cellular displacement trajectory during 4h for, left, a sub-confluent MDCK monolayer at low density, and right, the same monolayer at high density. Color code is the time. **b**) Log-log mean square displacement (MSD) evolution during jamming transition. n = 15 movies from N = 3 independent experiments. Color code is the moment in time. **c**) Left, cellular velocity field of sub-confluent MDCK monolayer at low density, right, same field at high density. Color code is the velocity magnitude. **d**) Average velocity magnitude during low and high cell density phase. 72 points, n = 24 movies from N = 5. p = 4e-25. [Scale bar 40 µm (a, c). P-value from two-sided Mann-Whitney U-test (d).]

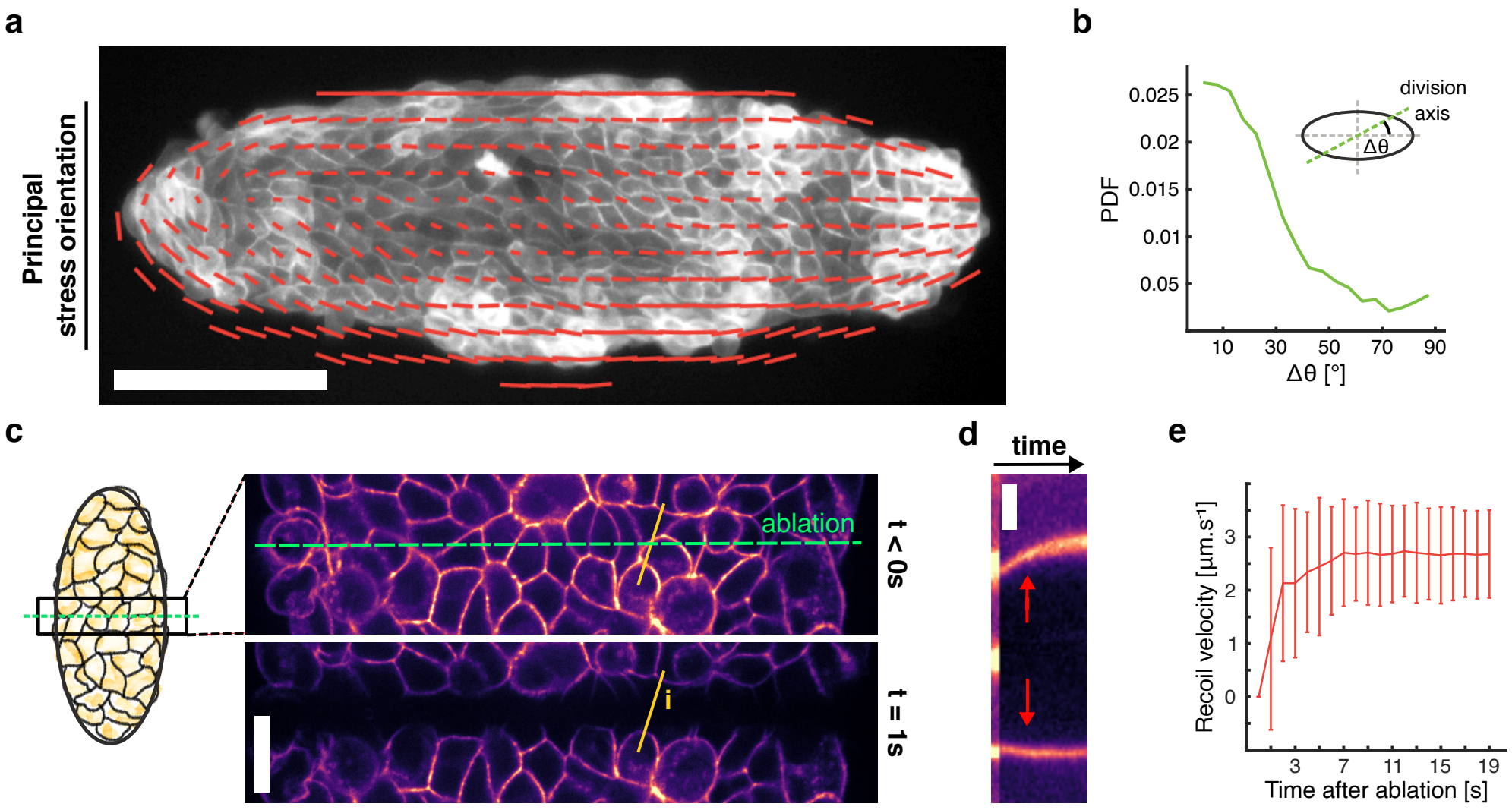

Fig. S6 Patterning induced stress anisotropy controls cell division orientation. **a**) CAAX GFP MDCK cells on ellipse shaped pattern. Principal stress orientation in red. **b**) Probability distribution function of the angle between cell division orientation and long axis of the ellipse. 228 cell divisions. n = 14 patterns from N = 2 independent experiments. **c**) CAAX GFP MDCK cells image focused on the center of the ellipse, right before and right after laser ablation across the small axis. **d**) Kymograph of cells temporal recoil after ablation, corresponding to the yellow line **i** in (c). **e**) Average (mean) evolution in time of the recoil velocity following laser ablation (happening at t = 0s). n = 14 patterns from N = 2 independent experiments. [Scale bar 125 µm (a), 15 µm (c), 2 µm (d).]

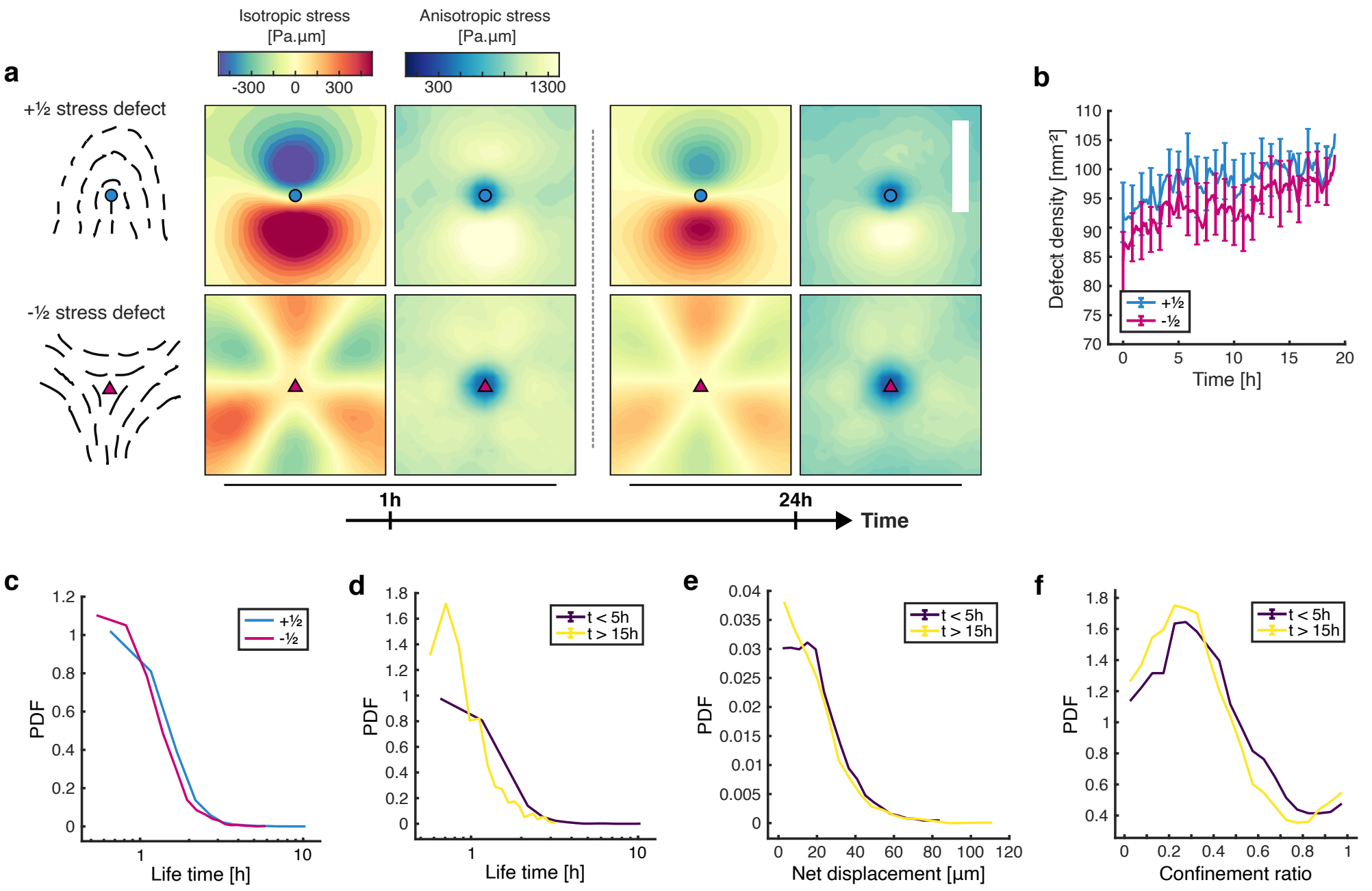

Fig. S7 Topological stress defects behavior in space and time is stable during densification. **a**) Isotropic (left) and anisotropic (right) stress field around stress topological defects oriented according to the schematic, during low and high density state. Top is $+\frac{1}{2}$ stress defects and bottom is $-\frac{1}{2}$ stress defects. Color code is the given stress component magnitude. 2441 $+\frac{1}{2}$ stress defects in low density state, 3152 $+\frac{1}{2}$ stress defects in high density state and 2346 $-\frac{1}{2}$ stress defects in low density state, 2894 $-\frac{1}{2}$ stress defects in high density state. n = 10 movies from N = 2 independent experiments. **b**) Average stress defects density evolution in time. n = 24 movies from N = 5 independent experiments. **c**) Probability distribution function (PDF) of one given stress defects life time in the tissue. n = 5 movies from N = 5 independent experiments. 2358 $+\frac{1}{2}$ stress defects and 1897 $-\frac{1}{2}$ stress defects. **d**) PDF of stress defects life time regardless of the charge for low and high density state. 839 stress defects tracked in the liquid phase and 644 stress defects tracked in the jammed phase. n = 5 movies from N = 5 independent experiments. **e**) PDF of stress defects net displacement regardless of the charge for low and high density state. 839 stress defects tracked in the liquid phase and 644 stress defects tracked in the jammed phase. n = 5 movies from N = 5 independent experiments. **f**) PDF of stress defects confinement ratio regardless of the charge for low and high density state. The confinement ratio ranges from 0 (confined or caged motion) to 1 (straight-line trajectory). 839 stress defects tracked in low density phase and 644 stress defects tracked in the high density phase. n = 5 movies from N = 5 independent experiments. [Scale bar 75 µm (a). All error bars show the confidence interval at 95 %.]

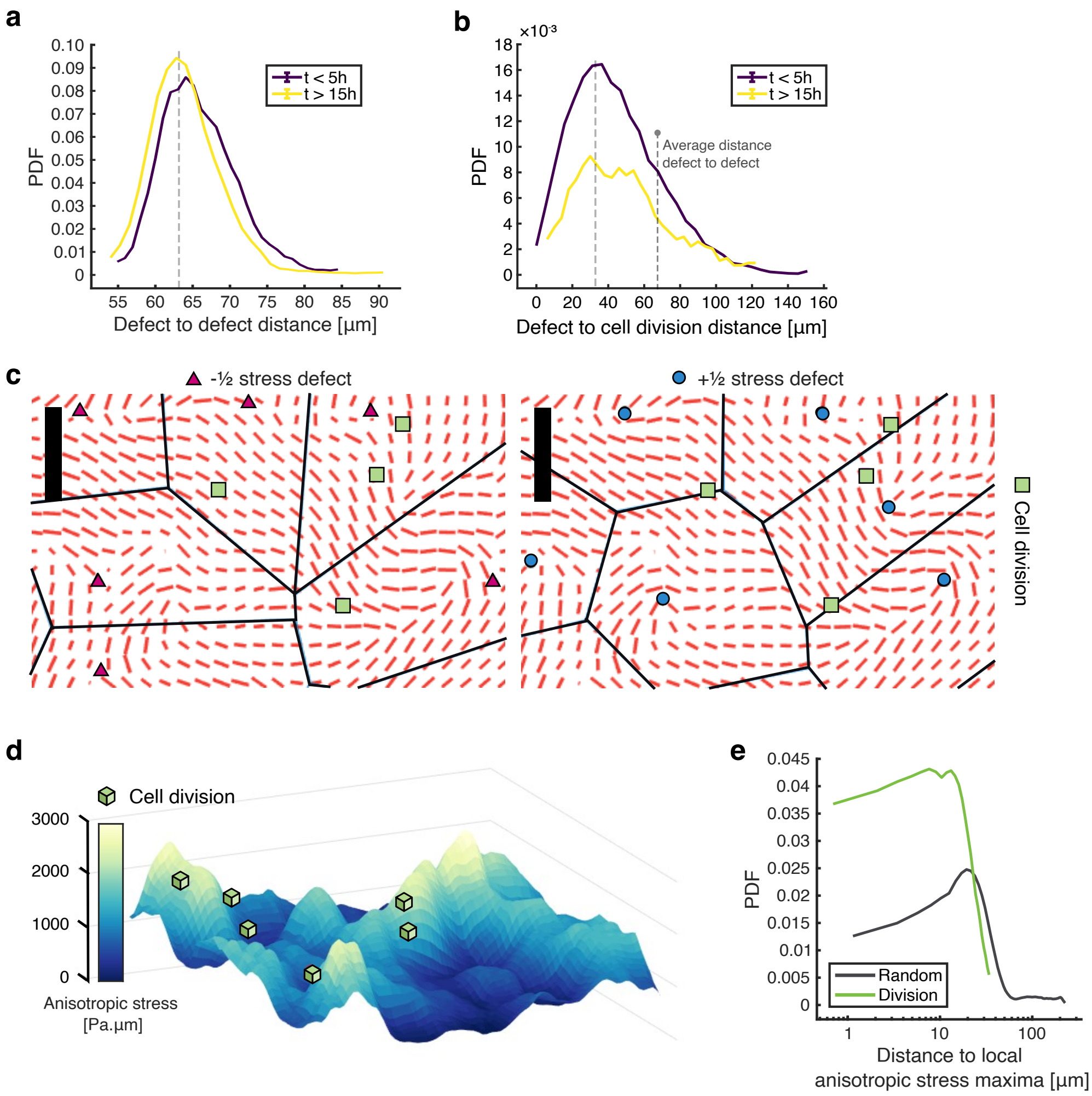

Fig. S8 Cell division events are localized away from stress topological defects. **a**) Probability density function (PDF) of average defect to defect distance in one given time (distances are computed on defects with identical charges) for low density and high density. 780 points (low density), 780 points (high density). n = 13 movies from N = 3 independent experiments. **b**) PDF of stress defects to cell division event distance regardless of the charge for liquid and jammed state. 676 divisions (low density), 270 divisions (high density). n = 13 movies from N = 3 independent experiments. **c**) Left, Voronoi tesselation using $-\frac{1}{2}$ stress defects (pink triangle) as generation cores. Right, Voronoi tesselation using $+\frac{1}{2}$ stress defects (blue circle) as generation cores. Principle stress orientation in red. Cell divisions in green square. The selected snapshot is representative of the typical situation during tissue densification (t = 5h). **d**) Mechanical anisotropic levels snapshot in the monolayer (t = 5h), represented as a mechanical potential landscape. Cell divisions (green square) are happening near local maxima. Color code is the anisotropic stress magnitude. **e**) PDF of distance to nearest anisotropic stress local maxima for cell divisions and randomly generated points in space and time. 1672 divisions, and 2219 randomly generated points. n = 13 movies from N = 3 independent experiments. [Scale bar 50 μm (c).]

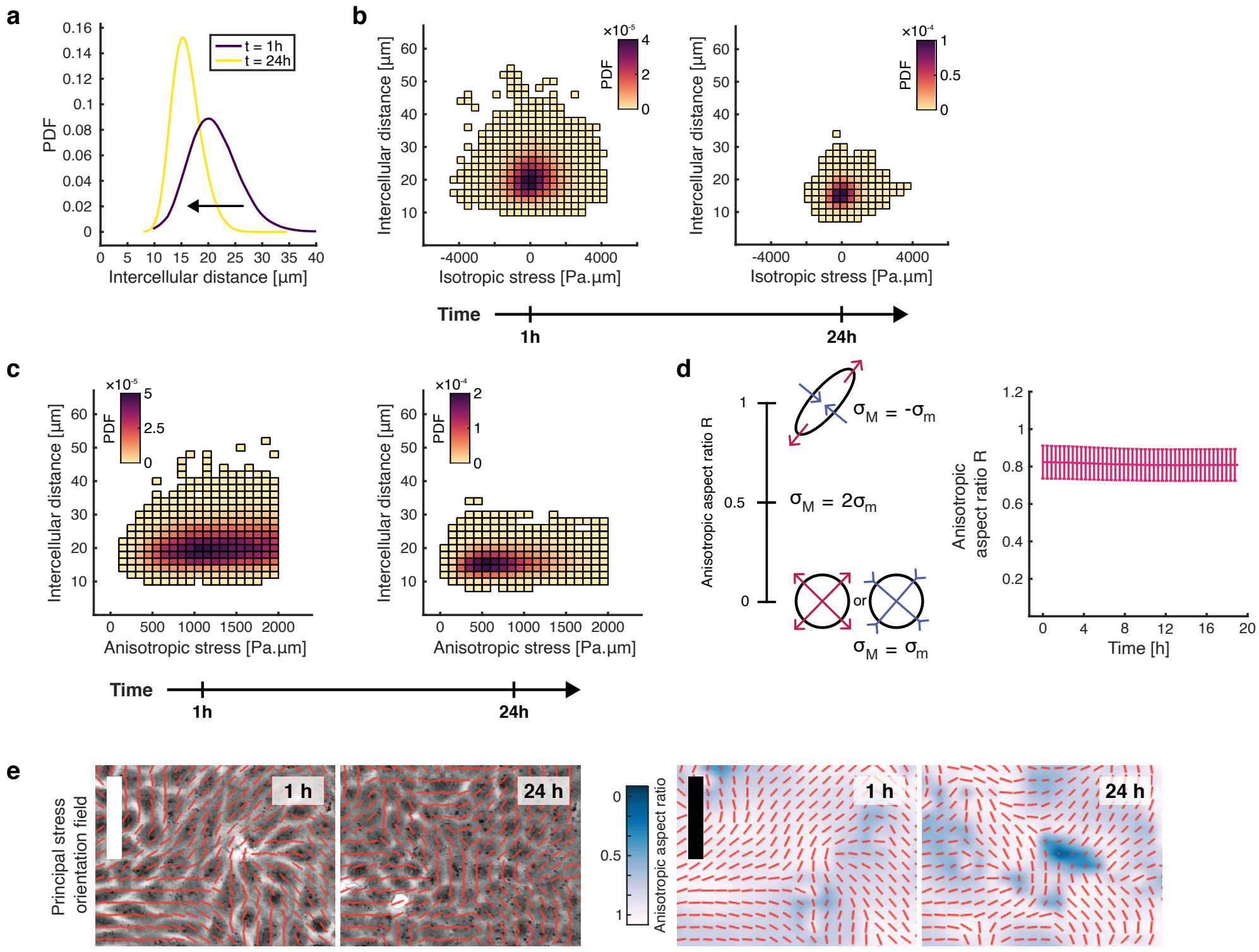

Fig. S9 Cell density and intercellular stress organization are coupled during tissue densification. **a**) Probability density function (PDF) of the distribution of intercellular distance between cells in the tissue in low density state and high density state. n = 15 movies from N = 3 independent experiments. **b**) 2D histogram of the isotropic stress and intercellular distance coupling within MDCK monolayer, left, at low density, and right, at high density. Color code is the PDF. n = 15 movies from N = 3 independent experiments. **c**) 2D histogram of the anisotropic stress and intercellular distance coupling within MDCK monolayer, left, at low density, and right, at high density. Color code is the PDF. n = 15 movies from N = 3 independent experiments. **d**) Right, simplified representation of the coupling between maximum and minimum stress for various value of anisotropic aspect ratio. Left, average evolution in time of the anisotropic aspect ratio within the whole tissue, for MDCK WT. n = 24 movies from N = 5 independent experiments. **e**) Right, phase contrast image of sub-confluent MDCK monolayer at low density, and at high density. Left, corresponding anisotropic aspect ratio field for low density and high density. Color code is the anisotropic aspect ratio value. Principal stress orientation is in red. [Scale bar 70 µm (e). All error bars show the confidence interval at 95 %.]

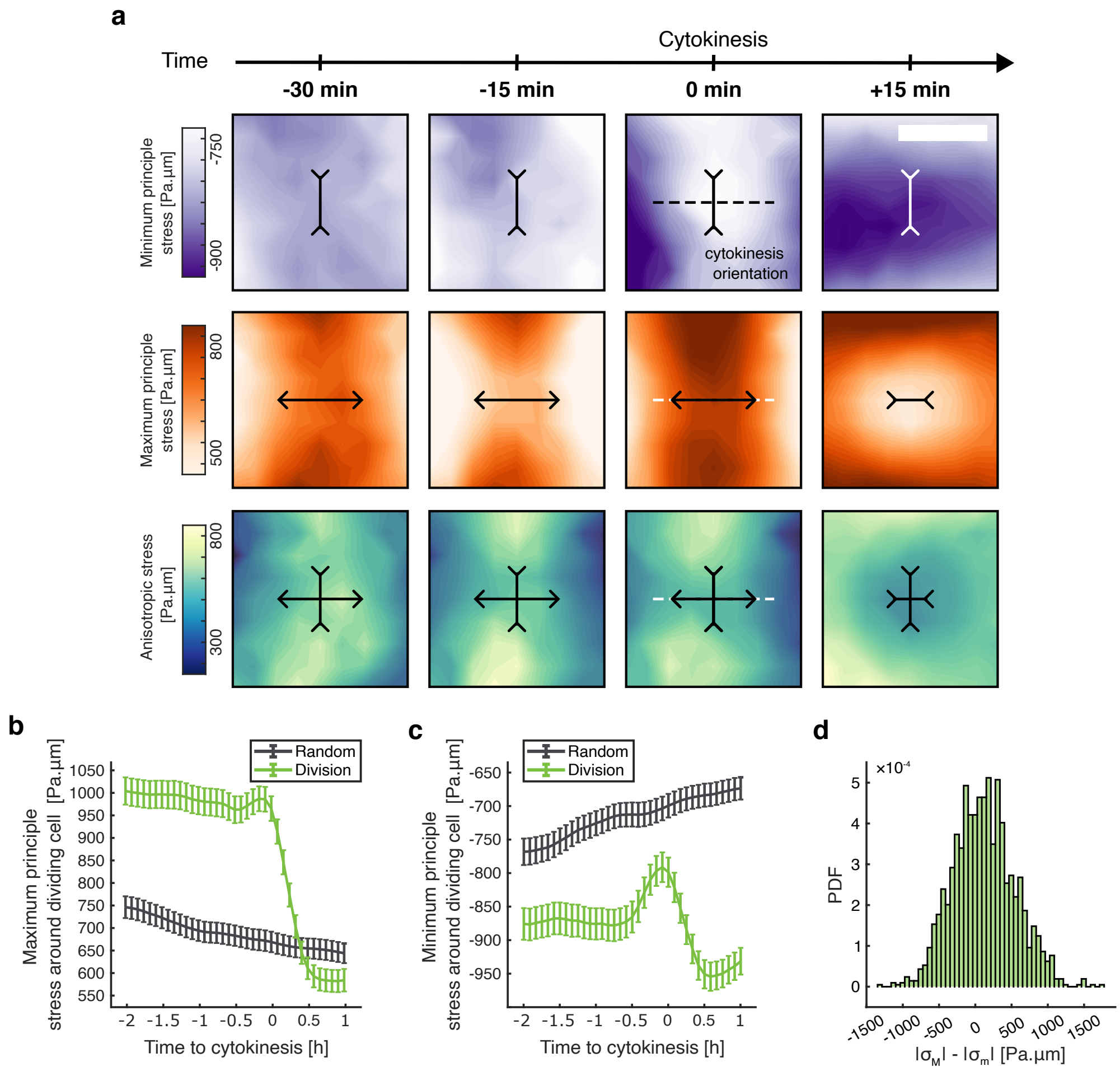

Fig. S10 The mechanical signature of cytokinesis is anisotropic. **a**) Stress field around cell division before and after cytokinesis, oriented along the cell division axis (left-right axis). The black double arrow show the orientation of the maximum and minimum stress, with their sign. Top, minimum principle stress, middle, maximum principle stress, and bottom anisotropic stress. 1478 cells division from n = 13 movies from N = 3 independent experiments. **b**) Average maximum principal stress around cell undergoing mitosis. Cytokinesis happens at t = 0h. 1672 cell divisions and 2219 random positions. n = 13 movies from N = 3 independent experiments. **c**) Average minimum principal stress around cell undergoing mitosis. Cytokinesis happens at t = 0h. 1672 cell divisions and 2219 random positions. n = 13 movies from N = 3 independent experiments. **d**) Probability distribution function (PDF) of the absolute contribution of maximum to minimum stress around cell division right before cytokinesis. 1672 cell divisions. n = 13 movies from N = 3 independent experiments. [Scale bar 25 µm (a). All error bars show the confidence interval at 95 %.]

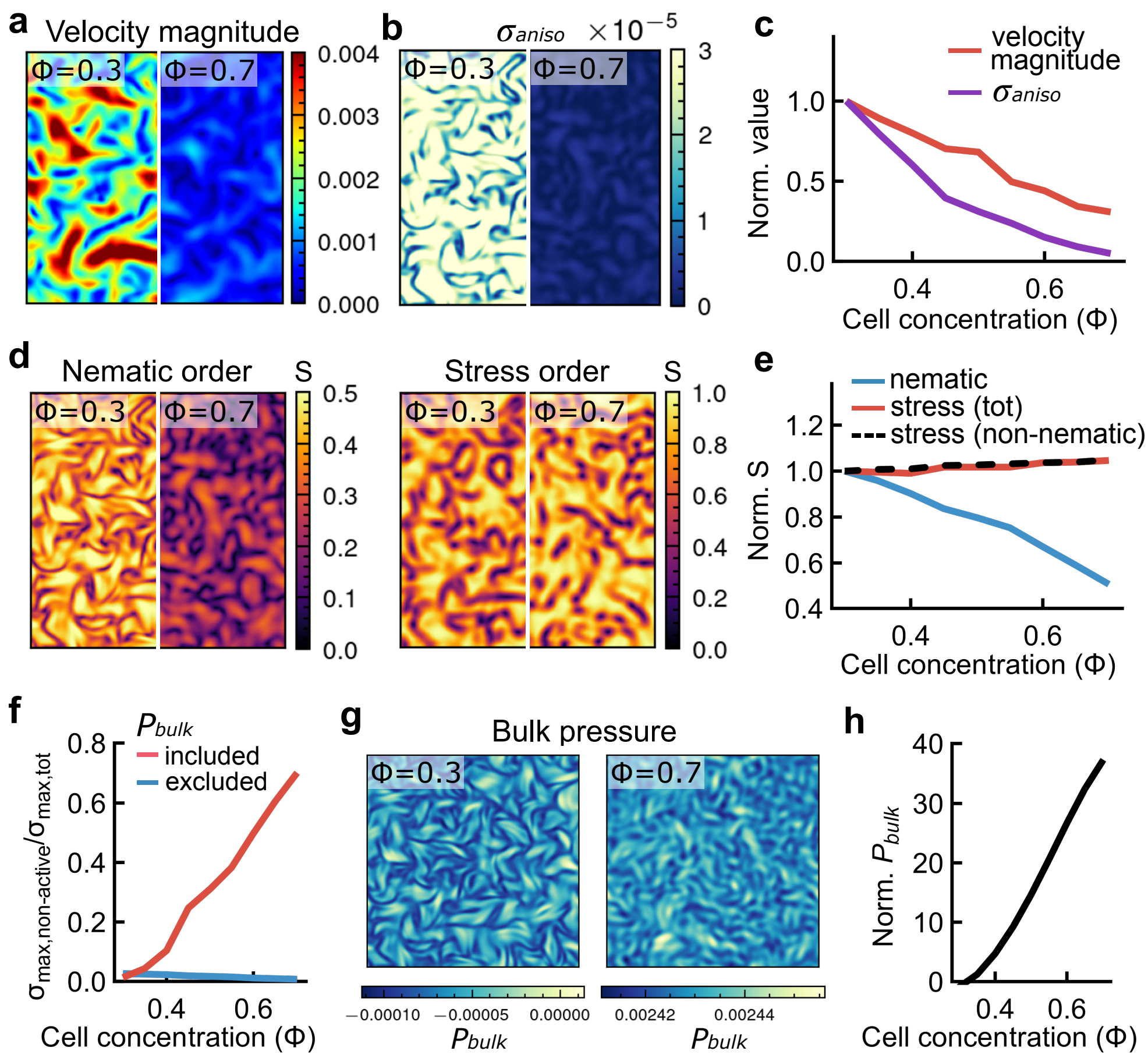

Fig. S11 Active nematic model with uniformly increased cell density reproduces key features of the tissue jamming transition. a, Velocity magnitude decreases with increasing cell concentration (ϕ). **b**, Anisotropic stress (σ_{aniso}) is progressively reduced at higher ϕ. **c**, Normalized velocity magnitude and anisotropic stress as functions of ϕ, both referenced to their respective values at $\phi = 0.3$, showing a coordinated decline with increasing density. **d**, Left: Nematic order collapses as ϕ increases. Right: In contrast, principal stress orientation remains robust. **e**, Normalized scalar order parameters for the nematic director (blue), principal stress (red), and principal stress calculated from the stress tensor with nematic stress excluded (black dashed), each referenced to their values at $\phi = 0.3$. While nematic order diminishes, stress and non-nematic stress alignment persists. **f**, Relative contribution of non-nematic stress to total stress, computed as the ratio of the maximal principal stress of non-nematic components ($\sigma_{\text{max,non-nematic}}$) to that of total stress ($\sigma_{\text{max,tot}}$), increases with ϕ (red). This trend disappears when bulk pressure (P_{bulk}) is excluded from the non-nematic stress (blue). **g,h)** Bulk pressure (P_{bulk}) rises with increasing cell concentration. [All simulations: $N = 3$; curves represent mean values.]

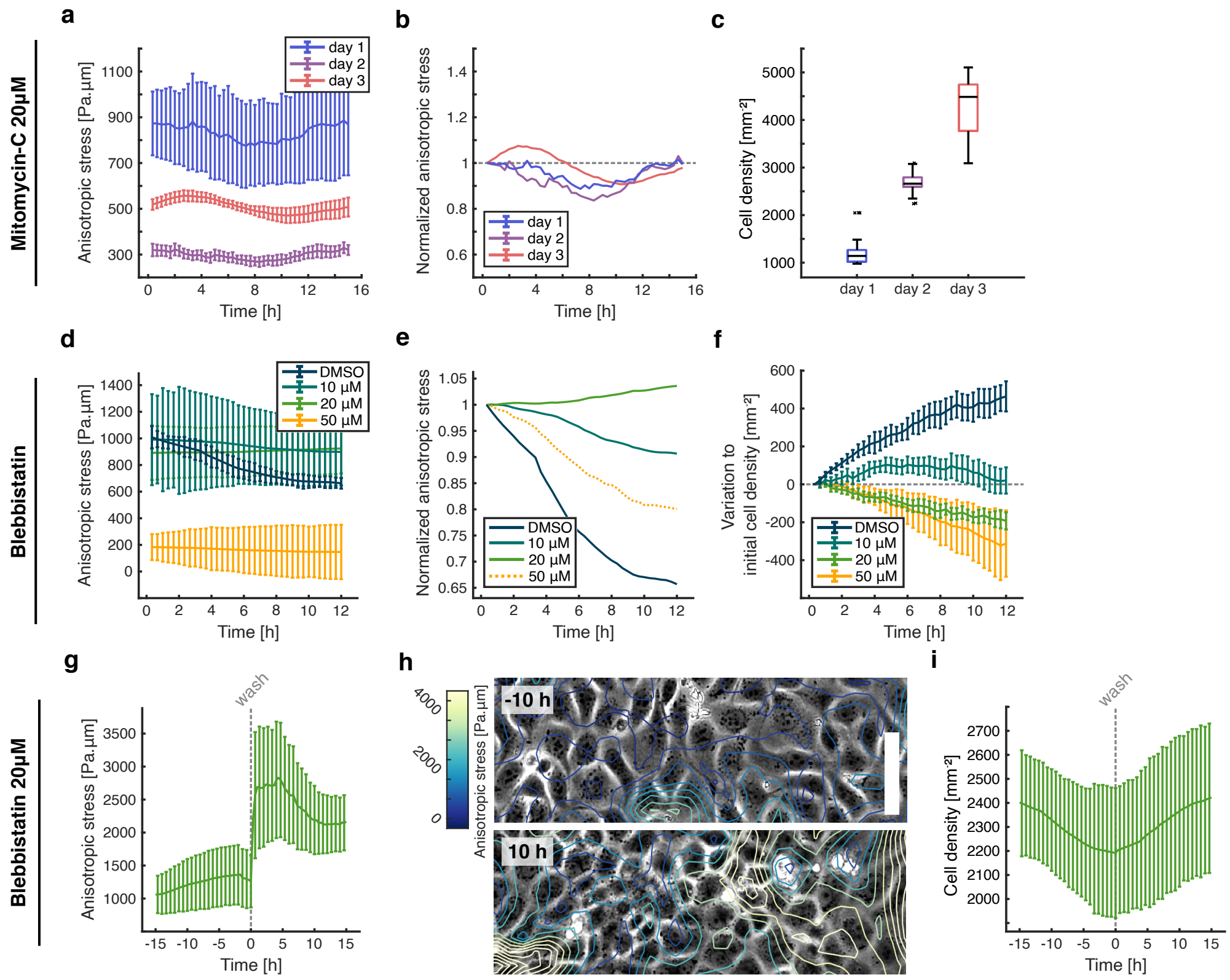

Fig. S12 Preventing cytokinesis freezes the anisotropic mechanical feedback loop. **a**) Mean anisotropic stress evolution for confluent MDCK monolayers under mitomycin-C added at various time steps after confluency was reached. n = 14 movies (added a day 1), n = 12 movies (added at day 2), n = 14 movies (added at day 3) from N = 3 independent experiments (day 1) and N = 2 independent experiments (day 2 & day 3). **b**) Mean normalized anisotropic stress evolution, corresponding to a). **c**) Average cell density for each mitomycin conditions, corresponding to a). **d**) Mean anisotropic stress evolution for confluent MDCK monolayers under various doses of blebbistatin, with DMSO as a control. n = 10 movies (10 μM), n = 10 movies (20 μM), n = 7 movies (50 μM), and n = 9 movies (DMSO) from N = 2 independent experiments. **e**) Mean normalized anisotropic stress evolution, corresponding to d). **f**) Mean cell density augmentation regarding the initial cell density in each experiments, corresponding to d). **g**) Mean anisotropic stress evolution for a confluent MDCK monolayer under 20 μM blebbistatin. A wash of the drug is done at t = 0h. n = 11 movies from N = 2 independent experiments. **h**) Phase contrast image of confluent MDCK monolayer, showing in overlap the local anisotropic stress field, before (t = -10h) and after (t = 10h) blebbistatin wash. **i**) Mean cell density during the course of the experiment corresponding to g). [Scale bar 50 µm (h). All error bars show the confidence interval at 95 %.]

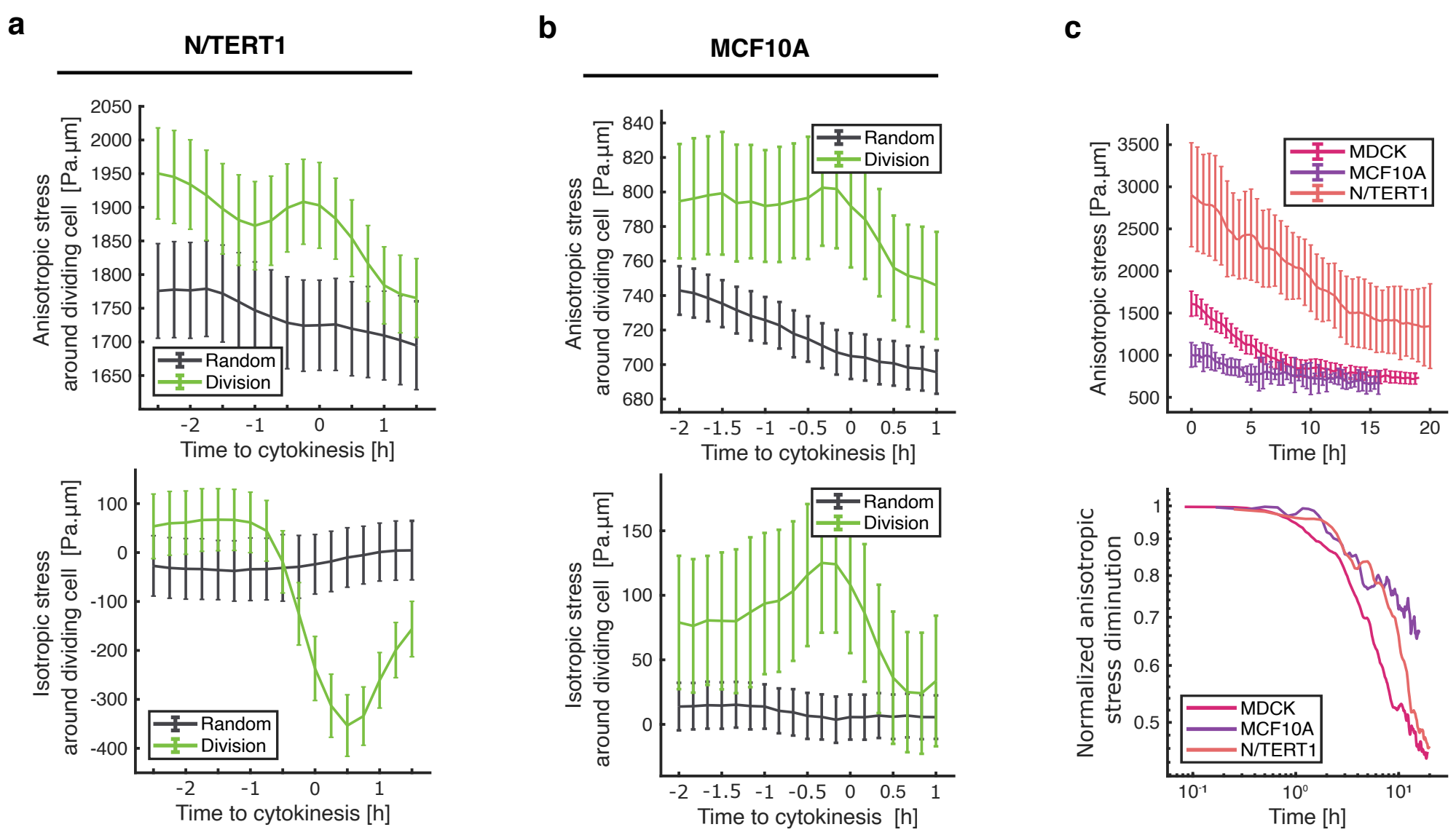

Fig. S13 Anisotropic stress and cell division feedback loop is recovered in different epithelial cell lines. **a**) N/TERT1 cells ; top, average anisotropic stress around cells undergoing mitosis, bottom, average isotropic stress around cells undergoing mitosis. Cytokinesis happens at t = 0h. 851 cell divisions and 704 random positions. n = 7 movies from N = 3 independent experiment. **b**) MCF10A cells ; top, average anisotropic stress around cells undergoing mitosis, bottom, average isotropic stress around cells undergoing mitosis. Cytokinesis happens at t = 0h. 354 cell divisions and 2348 random positions. n = 7 movies from N = 2 independent experiments. **c**) Top, average evolution in time of the anisotropic stress during jamming transition for various cell lines, and bottom, corresponding plot normalized by the maximum anisotropic stress levels of each cell types, in a log-log scale. n = 24 movies from N = 5 independent experiments (MDCK), n = 7 movies from N = 2 independent experiments (MCF10A) and n = 7 movies from N = 3 independent experiment (N/TERT1). [All error bars show the confidence interval at 95 %.]

Supplementary Table

Table 1 **A general mechanical principle for cell division across model system**. This table includes studies reporting cell division alignment with anisotropic stress-based cues, unless otherwise specified. When possible, we also report comparisons with cell-shape–based cues to ensure consistent evaluation across studies.

Author	System	Stress	Measure of stress	Source of stress	Shape cue
Lintilhac et al. (1984) [1]	Nicotiana tabacum pith tissue [1]	compressive	∅	**Ext**, Uniaxial compression	NA
Minc et al. (2011) [2]	Sea urchin zygote	compressive	∅	**Ext**, anisotropic CS	yes
Minc et al. (2011) [2]	Sea urchin zygote	compressive[2]	∅	**Ext**, isotropic CS	×
Fink et al. (2011) [3]	HeLa/RPE1 single cells	tensile	Laser ablation	**Ext**, anisotropic BC	yes
Fink et al. (2011) [3]	HeLa/RPE1 single cells	tensile[3]	Laser ablation	**Ext**, isotropic BC	×
LeGoff et al. (2013) [4]	Drosophila wing disc	tensile	Laser ablation	Self-organized	yes
Campinho et al. (2013) [5]	Zebrafish epiboly	tensile	Laser ablation	Self-organized	yes
Wyatt et al. (2015) [6]	MDCK suspended monolayer	tensile	Laser ablation	**Ext**, Uniaxial stretching	yes
Wyatt et al. (2015) [6]	MDCK suspended monolayer [*subset*]	tensile [4]	Laser ablation	**Ext**, Uniaxial stretching	yes
Bosveld et al. (2016) [7]	Drosophila pupal notum	mixed [5]	Laser ablation	Self-organized	yes
Louveaux et al. (2016) [8]	Arabidopsis thaliana shoot apex [1]	tensile	Laser ablation	Self-organized	NA
Hart et al. (2017) [9]	MDCK monolayer	tensile	∅	**Ext**, Uniaxial stretching	×
Finegan et al. (2018) [10]	Drosophila follicular epithelium	tensile	Laser ablation	Self-organized	×
Scarpa et al. (2018) [11]	Drosophila embryo, cell cycle 9 to 11	tensile	Laser ablation	Self-organized	×
Nestor-Bergmann et al. (2019) [12]	Xenopus tissue embryo	tensile	∅	**Ext**, Uniaxial stretching	yes
Nestor-Bergmann et al. (2019) [12]	Xenopus tissue embryo	mixed [6]	Vertex-based model	**Ext**, Uniaxial stretching	yes
Lisica et al. (2022) [13]	MDCK suspended monolayer	tensile	∅	**Ext**, Uniaxial stretching	yes
Lisica et al. (2022) [13]	MDCK suspended monolayer	compressive [7]	∅	**Ext**, Uniaxial compression	×
Blanchard et al. (2024) [14]	Drosophila embryo, cell cycle 14	compressive	Laser ablation	Self-organized	× [8]
Middelkoop et al. (2024) [15]	C. elegans + mouse zygote	compressive	∅	**Ext**, CS	yes

Footnotes appear on the following page.

∅ : not reported or not assessed in the original study. When the mechanical state was not assessed in the original study, we inferred it qualitatively based on the source of stress.

Ext : the internal stress organization is imposed by external constraints.

NA : Not applicable.

BC : Boundary conditions enforcing stress organization because of micropatterning.

CS : Compressive shell (or for Minc et al. micro-fabricated chambers).

[1]These studies were performed in plant epidermis rather than animal epithelia, but we considered them relevant because both systems share important features, including their organization as densely packed cellular sheets, and the existence of tissue-scale mechanical coupling. We chose to include them in this table because they report qualitatively similar couplings between anisotropic stress and division orientation, suggesting that the relationship identified here may extend beyond the specific epithelial contexts considered in this study.

[2]Uniaxial (z-axis) compressive confinement with isotropic in-plane geometry. The authors report cell division randomly oriented within the xy-plane but restricted to the plane perpendicular to compression.

[3]Isotropic tension generated by an isotropic pattern. Cells exhibit random in-plane division orientation under these conditions. Interestingly, external stretching of the pattern restores alignment of division orientation.

[4]In the subset (5%) of cells not aligning their division axis with the stretch direction, the authors show that cell long axis remains predictive of division orientation.

[5]The authors performed large-scale laser ablation and observed anisotropic recoil of the surrounding tissue.

[6]The authors report that division orientation is not determined by the global stretch orientation but correlates with local stress anisotropy and cell shape anisotropy.

[7]The authors report that under compressive loading, epithelial monolayers exhibit an increased fraction of out-of-plane divisions. This orientation is consistent with the axis of lowest compressive stress in a suspended monolayer and should correspond to maximal stress anisotropy direction.

[8]The authors report that high stress magnitude can override initial cell shape anisotropy.

Supplementary Notes

Invariants of the 2D stress tensor

The stress tensor $\boldsymbol{\sigma} = \begin{bmatrix} \sigma_{xx} & \sigma_{xy} \\ \sigma_{yx} & \sigma_{yy} \end{bmatrix}$ considered in this paper is treated as the 2D Cauchy stress tensor of continuum mechanics. The Cauchy stress tensor is symmetrical ($\sigma_{xy} = \sigma_{yx}$), and can be expressed in any system of coordinates using basis orientation transformation : $\boldsymbol{\sigma'} = \mathrm{D} \cdot \boldsymbol{\sigma} \cdot \mathrm{D}^T$ with $\mathrm{D} = \begin{bmatrix} \cos\theta & \sin\theta \\ -\sin\theta & \cos\theta \end{bmatrix}$, θ being the angle between the two systems of coordinates (x, y) and (x', y'). There are invariants associated with the stress tensor whose values do not depend on the coordinate system chosen. For the 2D stress tensor and using the Cayley–Hamilton theorem of linear algebra, first and second invariants can be defined as:

$$\mathrm{I}_1 = \mathrm{Tr}(\boldsymbol{\sigma}) = \sigma_{xx} + \sigma_{yy} \tag{1}$$

$$\mathrm{I}_2 = \det(\sigma) = \sigma_{xx}\sigma_{yy} - \sigma_{xy}^2 \tag{2}$$

In this paper, we consider the first invariant of the stress tensor I_1. I_1 quantifies the effect of mean normal stress at the point of space where the tensor is measured, therefore we define the isotropic stress as $\sigma_{iso} = \frac{\mathrm{I}_1}{2} = \frac{\sigma_{xx}+\sigma_{yy}}{2}$. $\sigma_{iso} > 0$ represents a case of local tension and $\sigma_{iso} < 0$ represents a case of local compression. Following the framework of continuum mechanics, $\boldsymbol{\sigma}$ can also be formalized as the sum of two sub-tensors:

$$\boldsymbol{\sigma} = \sigma_{iso}\mathbf{Id}_2 + \boldsymbol{\sigma_s}$$

$\boldsymbol{\sigma_s}$ is called the deviatoric stress tensor, and accounts for the non-isotropic part of the stress tensor. For the 2D deviator stress tensor, since $\boldsymbol{\sigma_s} = \boldsymbol{\sigma} - \sigma_{iso}\mathbf{Id}_2 = \begin{bmatrix} \frac{1}{2}(\sigma_{xx} - \sigma_{yy}) & \sigma_{xy} \\ \sigma_{yx} & \frac{1}{2}(\sigma_{yy} - \sigma_{xx}) \end{bmatrix}$, the first and second invariants can be expressed as:

$$\mathrm{J}_1 = \mathrm{Tr}(\boldsymbol{\sigma_s}) = 0 \tag{3}$$

$$\mathrm{J}_2 = -\det(\sigma_s) = [(\frac{\sigma_{xx} - \sigma_{yy}}{2})^2 + \sigma_{xy}^2] \tag{4}$$

One might observe that we compute J_2 with a minus sign comparing to I_2 but because σ_s is traceless, both $\det(\sigma_s)$ and $-\det(\sigma_s)$ are solution of the characteristic polynomial. As we want a solution with a physical meaning (in term of tensor norm), we choose the positive one. In this paper, we consider the second invariant of the deviatoric stress tensor : since σ_s is in a state of pure shear, J_2 quantifies the magnitude of the maximum shear applied. Because the dimension of J_2 is one that is a stress to the power of two, it is often convenient to define a second quantity μ by setting $\mu = \sqrt{\mathrm{J}_2}$. μ is sometimes referred to as maximum shear stress [16] and as deviatoric stress [17]: in this paper, we decide to call it anisotropic stress σ_{aniso}, following the convention of separating $\boldsymbol{\sigma}$ into two sub-components, one isotropic and one anisotropic. Even if σ_{iso} and σ_{aniso} are invariants, their expressions is still depending on σ_{xx}, σ_{xy} and σ_{yy}: in this paper, we choose to do our analysis in the coordinate system where $\sigma_{xy} = 0$. This can be simply achieved by diagonalizing $\boldsymbol{\sigma}$: we note $\boldsymbol{\sigma_d}$ the stress tensor equivalent to $\boldsymbol{\sigma}$ set in the basis where the shear stress is null. $\boldsymbol{\sigma_d}$ verifies:

$$\boldsymbol{\sigma_d} = \begin{bmatrix} \sigma_M & 0 \\ 0 & \sigma_m \end{bmatrix} \tag{5}$$

σ_M and σ_m are the two eigenvalues of $\boldsymbol{\sigma}$: they define two perpendicular orientations in which the shear stress is maximum and minimum. σ_M is called the maximum principle stress and σ_m is called the minimum principal stress. By construction, $\sigma_M > \sigma_m$ so the direction of application of σ_M is always the direction of the maximum

shear stress (Fig. S2f). In this paper, what we call the principal stress direction is the orientation of application of σ_M. Using the maximum and minimum principal stress, σ_{iso} and σ_{aniso} become:

$$\sigma_{iso} = \frac{\sigma_M + \sigma_m}{2} \tag{6}$$

$$\sigma_{aniso} = \frac{\sigma_M - \sigma_m}{2} \tag{7}$$

Stress topological defects

Topological defects are a universal class of spatial patterns found in multiple fields of physics [18–20]. Simply put, they can be defined as regions where a given parameter field collapses locally and can no longer be defined : this constitutes a topological singularity, or a topological defect. In this work, the existence of topological stress defects arises from the definition of the principal stress direction. In 2D, the relationship between $\boldsymbol{\sigma_d}$ and $\boldsymbol{\sigma}$ involves reorienting $\boldsymbol{\sigma}$ in a basis with orientation θ_p, where θ_p is the principal stress direction. We have: $\boldsymbol{\sigma_d} = \mathrm{D_p} \cdot \boldsymbol{\sigma} \cdot \mathrm{D_p}^T$ with $\mathrm{D_p} = \begin{bmatrix} \cos\theta_p & \sin\theta_p \\ -\sin\theta_p & \cos\theta_p \end{bmatrix}$

Projecting the equation, we have the system:

$$\begin{cases} \sigma_M = \sigma_{xx}\cos^2\theta_p + \sigma_{yy}\sin^2\theta_p + 2\sigma_{xy}\sin\theta_p\cos\theta_p \\ \sigma_m = \sigma_{xx}\sin^2\theta_p + \sigma_{yy}\cos^2\theta_p - 2\sigma_{xy}\sin\theta_p\cos\theta_p \\ 0 = (\sigma_{yy} - \sigma_{xx})\sin\theta_p\cos\theta_p + \sigma_{xy}(\cos^2\theta_p - \sin^2\theta_p) \end{cases}$$

This system of equation is usefull to express σ_M, σ_m and θ_p only using a function of $(\sigma_{xx}, \sigma_{yy}, \sigma_{xy})$. In particular, the expression concerning θ_p is :

$$\tan 2\theta_p = \frac{2\sigma_{xy}}{(\sigma_{xx} - \sigma_{yy})} \tag{8}$$

From here, one can see directly that this equation is not defined when $(\sigma_{xx} - \sigma_{yy)} \rightarrow 0$. By definition, any local region where this condition is approached constitutes a topological defect, as θ_p becomes undefined. Since this property holds in any basis, it is natural that topological defects theoretically emerge in regions where $\sigma_{\text{aniso}} \rightarrow 0$.

It is important to emphasize that in this paper, we do not define stress topological defects based on this analytical condition. Instead, we compute the topological charge using a winding number approach (see Methods). Through this method, we experimentally recover the natural mathematical property expressed in equation 8.

Supplementary References

[1] Lintilhac, P.M., Vesecky, T.B.: Stress-induced alignment of division plane in plant tissues grown in vitro. Nature **307**(5949), 363–364 (1984)

[2] Minc, N., Burgess, D., Chang, F.: Influence of cell geometry on division-plane positioning. Cell **144**(3), 414–426 (2011)

[3] Fink, J., Carpi, N., Betz, T., Bétard, A., Chebah, M., Azioune, A., Bornens, M., Sykes, C., Fetler, L., Cuvelier, D., *et al.*: External forces control mitotic spindle positioning. Nature cell biology **13**(7), 771–778 (2011)

[4] LeGoff, L., Rouault, H., Lecuit, T.: A global pattern of mechanical stress polarizes cell divisions and cell shape in the growing drosophila wing disc. Development **140**(19), 4051–4059 (2013)

[5] Campinho, P., Behrndt, M., Ranft, J., Risler, T., Minc, N., Heisenberg, C.-P.: Tension-oriented cell divisions limit anisotropic tissue tension in epithelial spreading during zebrafish epiboly. Nature cell biology **15**(12), 1405–1414 (2013)

[6] Wyatt, T.P., Harris, A.R., Lam, M., Cheng, Q., Bellis, J., Dimitracopoulos, A., Kabla, A.J., Charras, G.T., Baum, B.: Emergence of homeostatic epithelial packing and stress dissipation through divisions oriented along the long cell axis. Proceedings of the National Academy of Sciences **112**(18), 5726–5731 (2015)

[7] Bosveld, F., Markova, O., Guirao, B., Martin, C., Wang, Z., Pierre, A., Balakireva, M., Gaugue, I., Ainslie, A., Christophorou, N., *et al.*: Epithelial tricellular junctions act as interphase cell shape sensors to orient mitosis. Nature **530**(7591), 495–498 (2016)

[8] Louveaux, M., Julien, J.-D., Mirabet, V., Boudaoud, A., Hamant, O.: Cell division

plane orientation based on tensile stress in arabidopsis thaliana. Proceedings of the National Academy of Sciences **113**(30), 4294–4303 (2016)

[9] Hart, K.C., Tan, J., Siemers, K.A., Sim, J.Y., Pruitt, B.L., Nelson, W.J., Gloerich, M.: E-cadherin and lgn align epithelial cell divisions with tissue tension independently of cell shape. Proceedings of the National Academy of Sciences **114**(29), 5845–5853 (2017)

[10] Finegan, T.M., Na, D., Cammarota, C., Skeeters, A.V., Nádasi, T.J., Dawney, N.S., Fletcher, A.G., Oakes, P.W., Bergstralh, D.T.: Tissue tension and not interphase cell shape determines cell division orientation in the drosophila follicular epithelium. The EMBO journal **38**(3), 100072 (2019)

[11] Scarpa, E., Finet, C., Blanchard, G.B., Sanson, B.: Actomyosin-driven tension at compartmental boundaries orients cell division independently of cell geometry in vivo. Developmental cell **47**(6), 727–740 (2018)

[12] Nestor-Bergmann, A., Stooke-Vaughan, G.A., Goddard, G.K., Starborg, T., Jensen, O.E., Woolner, S.: Decoupling the Roles of Cell Shape and Mechanical Stress in Orienting and Cueing Epithelial Mitosis. Cell Reports **26**(8), 2088–21004 (2019)

[13] Lisica, A., Fouchard, J., Kelkar, M., Wyatt, T.P., Duque, J., Ndiaye, A.-B., Bonfanti, A., Baum, B., Kabla, A.J., Charras, G.T.: Tension at intercellular junctions is necessary for accurate orientation of cell division in the epithelium plane. Proceedings of the National Academy of Sciences **119**(49), 2201600119 (2022)

[14] Blanchard, G.B., Scarpa, E., Muresan, L., Sanson, B.: Mechanical stress combines with planar polarised patterning during metaphase to orient embryonic epithelial cell divisions. Development **151**(10), 202862 (2024)

[15] Middelkoop, T.C., Neipel, J., Cornell, C.E., Naumann, R., Pimpale, L.G., Jülicher, F., Grill, S.W.: A cytokinetic ring-driven cell rotation achieves hertwig's rule in early development. Proceedings of the National Academy of Sciences **121**(25), 2318838121 (2024)

[16] Tambe, D.T., Corey Hardin, C., Angelini, T.E., Rajendran, K., Park, C.Y., Serra-Picamal, X., Zhou, E.H., Zaman, M.H., Butler, J.P., Weitz, D.A., *et al.*: Collective cell guidance by cooperative intercellular forces. Nature materials **10**(6), 469–475 (2011)

[17] Gauquelin, E., Kuromiya, K., Namba, T., Ikawa, K., Fujita, Y., Ishihara, S., Sugimura, K.: Mechanical convergence in mixed populations of mammalian epithelial cells. The European Physical Journal E **47**(3), 21 (2024)

[18] Vromans, A.J., Giomi, L.: Orientational properties of nematic disclinations. Soft matter **12**(30), 6490–6495 (2016)

[19] Fumeron, S., Berche, B.: Introduction to topological defects: from liquid crystals to particle physics. The European Physical Journal Special Topics **232**(11), 1813–1833 (2023)

[20] Fardin, M.-A., Ladoux, B.: Living proof of effective defects. Nature Physics **17**(2), 172–173 (2021)
