# Supplementary material for: A principal-stress rule for cell division in epithelia
Source: Nat Commun. 2026 Sep 23;17:11062. doi: 10.1038/s41467-026-77813-5 (PMC13691091; doi:10.1038/s41467-026-77813-5)
Supplement: Supplementary file 3 [file 41467_2026_77813_MOESM2_ESM.pdf]

Description of Additional Supplementary Files

Supplementary Movie 1: Timelapse video of densification of MDCK WT cells. Frame rate 5 min. Nematic director field in blue. Scale bar 80 μm.

Supplementary Movie 2: Timelapse video of densification of MDCK WT cells, with the corresponding isotropic and anisotropic stress field. Frame rate 5 min. Principal stress orientation field in red. Scale bar 80 μm.

Supplementary Movie 3: Timelapse video of intestinal organoid-derived 2D monolayer, after three days culture, with villin tomato channel. Principal stress orientation field in red. Frame rate 10 min. Scale bar 80 μm.

Supplementary Movie 4: Timelapse video of densification of MDCK WT cells, corresponding to Movie S1. Frame rate 5 min. Principal stress orientation field in red. Scale bar 80 μm.

Supplementary Movie 5: Timelapse video of densification of MDCK FUCCI cells. Frame rate 5 min. Magenta is G1/G0 signal, green is S-G2-M signal, overlapped with phase contrast images. Scale bar 80 μm.

Supplementary Movie 6: Timelapse video of densification of MCF10A cells, with the corresponding isotropic and anisotropic stress field. Frame rate 10 min. Principal stress orientation field in red. Scale bar 80 μm.

Supplementary Movie 7: Timelapse video of densification of N/TERT1 cells, with the corresponding isotropic and anisotropic stress field. Frame rate 15 min. Principal stress orientation field in red. Scale bar 80 μm.
